# Growing kalo (taro) to promote culture and health in the Continental US

**DOI:** 10.3389/fpubh.2025.1689052

**Published:** 2025-10-10

**Authors:** Leialoha Ka’ula, Nicole lee Kamakahiolani Ellison, Constance James, Ashley Oshiro, Kacyn Ideue, Kahoku Ka’ula, Alexandra Malia Jackson

**Affiliations:** ^1^Ka ‘Aha Lahui O ‘Olekona, Beaverton, OR, United States; ^2^Public Health, Pacific University, Forest Grove, OR, United States; ^3^Biology, Pacific University, Forest Grove, OR, United States

**Keywords:** Native Hawaiian, food sovereignty, diaspora, community garden, traditional food

## Abstract

**Introduction:**

A growing number of Native Hawaiians live in the continental US. Without access to the ‘āina (land) in Hawai’i, māla kalo (community gardens used to grow taro) may offer a space for these communities to increase access to traditional foods and create community connections.

**Methods:**

We formed a community–research hui to engage in a community-based participatory research process to explore potential benefits of a māla kalo. We used an explanatory mixed-methods Indigenous evaluation approach, including a survey and interview with program volunteers and leaders to identify implementation strategies and thematic analysis to explore potential benefits of a māla kalo on the continent.

**Results:**

A total of 12 participants and 5 program leaders, aged 18 to 75 years, completed a survey and interview at the end of the 2023 growing season. The findings suggested high levels of satisfaction and an interest in participating more frequently. Qualitative data suggested that volunteering at the māla kalo may support connections to self, community, and land, learning and sharing of knowledge, and connection to culture.

**Conclusion:**

Community gardens that grow traditional foods may foster relationships, health, and culture within a displaced Indigenous community. Future steps should include continued evaluation of the health benefits of community gardens that grow traditional foods using culturally relevant measures and infrastructure development to create resources that support other organizations in scaling up similar programs.

## Introduction

Prince Jonah Kuhio Kalanianaʻole believed the only way to rehabilitate the Native Hawaiian people was through the ʻāina (land that feeds). Prior to Western contact, Native Hawaiians had a sophisticated, symbiotic food system that provided the necessary resources for the ʻāina and people to thrive. The ʻāina is the origin, mother, inspiration, and environment of the Native Hawaiian people (1), with kalo (taro) as the origin of life. In the Kumulipo (Hawaiian creation chant), Hāloanakalaukapalili was stillborn and buried in the ʻāina (land) by Papahānaumokuākea at the request of Hoʻohōkūkalani. Born from this child in the ʻāina is kalo, a plant that nourished the second-born son, Hāloa, from whom Native Hawaiians trace their lineage (2).

Culturally grounded and land-based food sovereignty interventions hold promise for promoting health within Native Hawaiian and other Indigenous communities. Culturally grounded interventions are rooted in cultural practices, values, beliefs, and ways of knowing (3, 4). These interventions uplift the strengths of communities as a means to promote health and prevent disease. Culturally grounded interventions have been effective in reducing substance use in young adults, preventing and managing diabetes, and controlling hypertension (5–9). Alternatively, non-culturally grounded, evidence-based interventions may lack contextual fit and, therefore, be less effective than those that are built upon existing community strengths (10, 11). Similar to culturally grounded interventions, land-based interventions specifically honor the relationship between Indigenous people and the land, as well as the cultural practices associated with the land, to improve health outcomes (12). A recent systematic review of land-based interventions indicated potential benefits in community engagement, as well as spiritual, physical, emotional, and mental health outcomes (12). Lastly, promoting food sovereignty, or the right to access healthy and culturally appropriate foods and to define their own food and agriculture systems (13, 14), is central to promoting health and wellbeing in Indigenous communities (15).

Both culturally grounded and land-based initiatives illustrate the importance of connectedness—to place, community, culture, and family—as described in the Indigenous Connectedness Framework (16). The Indigenous Connectedness Framework centers on connection and relationships rather than on individuals’ physical health. This framework has also been used as the foundation for a conceptual framework describing potential health outcomes of food sovereignty initiatives (15). It describes connection and relationships as mechanisms through which food sovereignty activities (e.g., sharing food and food knowledge and caring for the land) have proximal effects on health-related outcomes, such as self-efficacy for healthy eating and increased knowledge of traditional foods, and distal effects on wellbeing.

While the Indigenous Connectedness Framework and the conceptual framework of potential effects of Indigenous food sovereignty apply broadly to Indigenous people and cultures, one particularly important aspect for Native Hawaiian communities is the description of the deep connection to the land. “Āina” is commonly translated as land; however, ʻāina” means “the land that feeds,” emphasizing the reciprocal and relational connection between the land and those who live on it. This reciprocal relationship is also illustrated in a Hawaiian proverb, ʻŌlelo Noʻeau #531 “He aliʻi ka ʻāina; He kauā ke kanaka. The land is chief; People are its servant.” By defining the land as feeding the community, it highlights the kuleana (responsibility) and reciprocity involved in caring for the land, with the understanding that, in return, the land will care for and feed the people (17).

According to the 2020 United States (US) Census, more Native Hawaiians live away from Hawaiʻi than on the islands (18). As Native Hawaiians continue to leave Hawaiʻi at an increasing rate, finding community and connection to the land away from home can be an opportunity to promote health. In 2021, the Ka ʻAha Lāhui O ʻOlekona Hawaiian Civic Club (KALO HCC), a community-based organization that aims to promote health, education, and culture among Native Hawaiians living in the Pacific Northwest, recognized the importance of connection to the land and kalo and worked with a regional food bank to create a māla kalo (garden used to grow taro). Kalo was chosen as the focus of the garden due to its spiritual significance and role as a traditional food. All parts of the kalo plant, a primary food within the ahupuaʻa and a staple of the traditional diet, can be consumed (19, 20). The corm is pounded into paʻiʻai, which is then mixed with water to make poi, while the stem and leaves are steamed to prepare traditional dishes such as lau lau and squid luʻau (19, 20).

**Figure 1:**
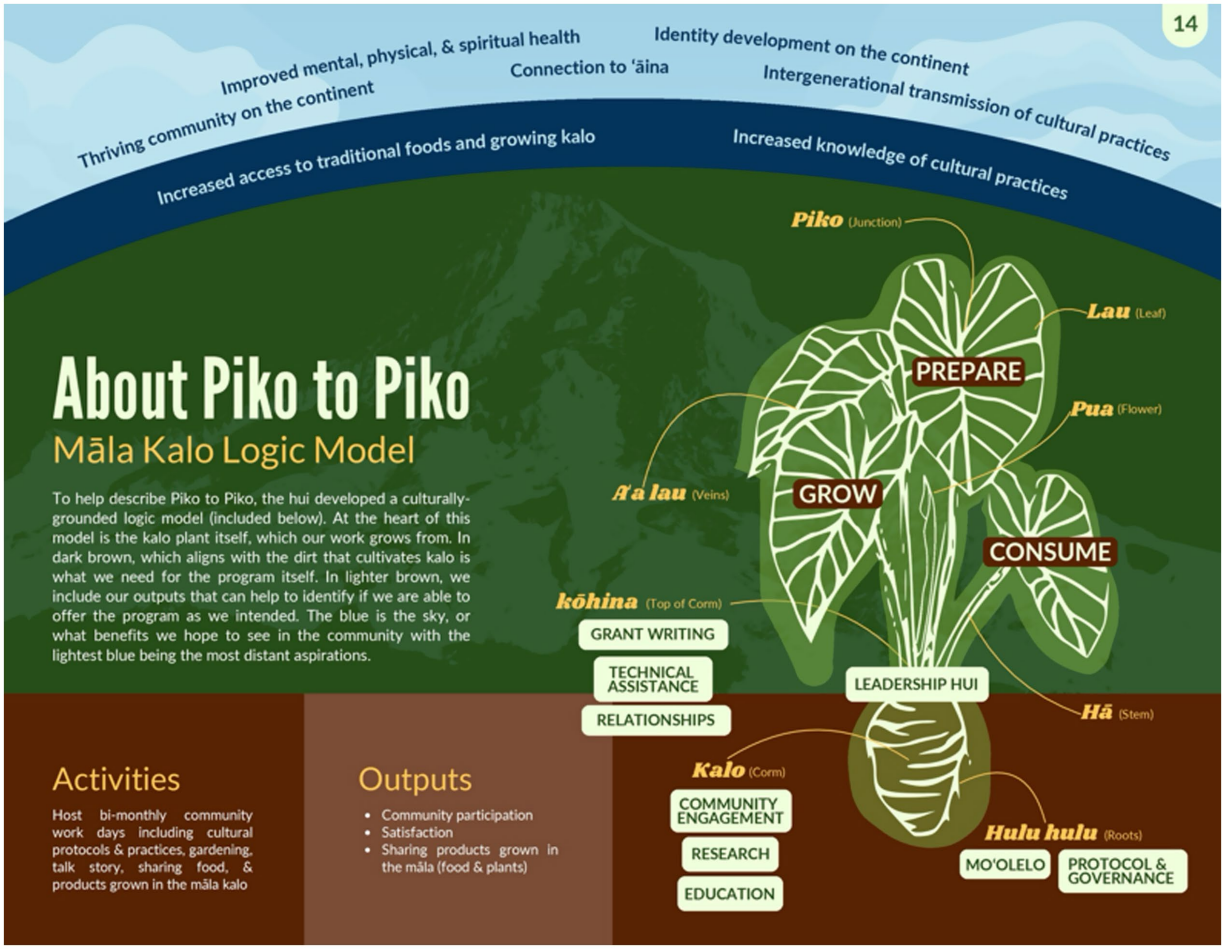
Māla Kalo logic model.

During the 2021 growing season, the KALO HCC focused on learning how to grow kalo in Oregon, which has a much shorter growing season (March to November) and a substantially different climate compared to Hawaiʻi. After confirming that kalo could be successfully grown in Oregon, the māla kalo expanded in size (from 24 to 960 square feet) and reach (to over 100 volunteers each season) (21). During the 2023 growing season, the KALO HCC facilitated weekly workdays one to two times per month with 1 to 2 participants, as well as monthly workdays with an average of 10–20 participants (approximately 60 total workdays during the growing season). During monthly workdays, families came together to follow cultural protocols; learn Hawaiian values, practices, and language; share knowledge of growing kalo; maintain the garden; talk story (talk informally and share stories); work together in the soil; and enjoy a meal. Community members were encouraged to take home products grown in the garden (e.g., small plants or edible leaves) when available. A logic model based on the kalo plant and Native Hawaiian values and practices that describes this initiative is included in Figure 1.

As culturally grounded and land-based initiatives show promise in promoting the health of Indigenous communities, to understand the potential benefits of growing kalo in a community garden on the continent, we used a community-based participatory approach (CBPR), including establishing a community–research hui (team), to conduct a preliminary Indigenous mixed-methods evaluation of growing kalo on the continent. The evaluation focused on two aims: (1) assessing program implementation strategies (i.e., process evaluation) and (2) conducting an exploratory outcome evaluation to identify the benefits of a community garden for growing kalo on the continent. The implementation strategies assessed included participation, satisfaction, and products received from the māla kalo. As this is a new program, an exploratory analysis of the qualitative data collected was conducted to identify the potential benefits of a māla kalo on the continent, as reported by program leaders and participants.

## Methods

We employed a CBPR approach, in which the members of the community-research hui worked together on all aspects of this project, from digging in the dirt at the māla kalo to data collection, analysis, and reporting. The hui (all of whom volunteered their time) met monthly to discuss measures used, study design, and analyses. The community identified the needs and goals of the evaluation, while the research team served as consultants, navigating the process from the development of data collection measures to analysis and dissemination. All decisions were made as a hui, with the underlying recognition that this project provides information to support the displaced Native Hawaiian community. As an exploratory Indigenous evaluation, we centered strengths of the community, Indigenous knowledge, and culture throughout the study design, data collection, and analysis processes (22). The evaluation was guided by the eight phases of Māʻawe Pono, which include the following: ʻimi naʻauao (search for wisdom), hoʻoliuliu (preparation of the project), hailona (pilot testing), hoʻoluʻu (immersion), hoʻomōhala (incubation), haiʻiloaʻa (articulation of solution(s)), hōʻike (demonstration of knowledge), and kūkulu kumuhana (pooling of strengths) (23). All findings were shared with the participants and organization members prior to external dissemination by email, during a workday, and at an organization-wide meeting. Each participant received an email containing the community report and a request to provide feedback. The participants did not provide any feedback. The preliminary findings were shared after a māla kalo workday, and the community report (Appendix A) was shared during an in-person meeting with 102 members of the community organization. No changes were requested during or after either meeting. Community members expressed appreciation for the hui’s commitment to sharing the findings with them before broad distribution.

At the end of the 2023 growing season (November – December), māla kalo volunteers aged 18 years and older were invited by email to complete an online survey and participate in a Zoom interview. The survey took approximately 15 min to complete, and the interview lasted approximately 30 min. The volunteers were compensated with a $30 gift card. Five program leaders who were employees or board members of the organization and assisted with the development and implementation of this program were also invited to complete a 5-min survey and a 60-min interview and were compensated with a $50 gift card. This study was certified as exempt by the Pacific University Institutional Review Board. All participants provided written consent at the start of the survey and oral consent at the beginning of the interview.

### Positionality

The authors come from diverse backgrounds, including Native Hawaiian heritage, origins in Hawaiʻi, and training in public health, nutrition, and education, along with traditional knowledge of Hawaiian culture. They were raised in Hawaiʻi and on the continent, and all authors currently live away from the islands. The experiences and expertise of the authors were complementary. Kuleana (responsibility) statements, which outline the positionality of the hui, are included in the community report (Appendix A, pages 7–9).

### Quantitative measures

Demographic data, including sex, age, primary ethnicity, marital status, number of adults in the household, education level, income, and employment status, were collected from the participants. To maintain confidentiality, marital status, household size, education level, and household income were not collected from the program leaders. The participants and program leaders were asked to indicate how frequently they volunteered during the 2023 March–November growing season (response categories included the following: 1–2, 3–4, 5–6, 7–8, and more than 8). Satisfaction was measured using a single question on a 5-point scale ranging from extremely satisfied to extremely dissatisfied. The volunteer surveys were longer than the program leader surveys, as they included questions about social connection and stress, which were not included in this analysis due to the small sample size and hui concerns of confidentiality.

### Qualitative measures

The interview protocol (Appendix B) included questions about possible improvements to participating in the māla kalo, what the participants appreciated about working at the māla kalo, and what they learned from their involvement. The program leaders’ interviews mirrored those of the volunteers, with two key differences: (1) they included additional questions about reaching the community and encouraging participation and (2) questions were framed to ask leaders about the potential effects of the māla kalo on volunteers. The interview protocol was pilot tested with three research–community hui members who completed the protocol with one another prior to implementation; no changes were made. All interviews were conducted via Zoom by the members of the hui, audio recorded, and transcribed by three authors. All transcriptions were reviewed by the last author.

### Analysis

Descriptive statistics were analyzed using SPSS (version 29, Armonk NY, 2024). Due to the small sample size, we present preliminary descriptive data, including sociodemographic data and ratings of participation, satisfaction, and products grown. Qualitative data were analyzed using reflexive thematic analysis (24) and MAXQDA 2022 (Berlin, 2022). First, one author reviewed the transcripts multiple times, taking notes and discussing findings with the other authors, leading to an initial codebook and coding schema. The initial codes included, but were not limited to, feedback about the māla, relationships/pili, community, the significance of kalo, and identity. Next, two authors used MAXQDA (2022) to code the transcripts using the initial coding schema. The codes, codebook, and application of the codes (i.e., when the codes were used) were then discussed with the authorship team, and the transcripts were reviewed and re-coded. The codes were then grouped into patterns or themes in alignment with the evaluation questions (1) to assess implementation strategies and (2) to describe potential benefits of growing kalo on the continent.

To evaluate implementation strategies, quantitative and qualitative data were integrated into a joint display for each domain (e.g., satisfaction), as outlined by Aschbrenner et al. (25). This process included identifying implementation domains, aligning the quantitative and qualitative sources for each domain, generating aggregated data for each strand, integrating analyses using joint displays, and drawing meta-inferences from both strands of data collected (25). A mixed-methods approach with a joint display was used to allow for the presentation of the quantitative and qualitative data separately, which were then merged into a meta-inference, comparing, contrasting, and expanding on the findings from each strand of the data collected (26–28).

As the codes were grouped into themes describing the benefits of growing kalo, we used the Indigenous Connectedness Framework as a guiding model, recognizing the close parallels with the four domains of connection: environmental, family, community, and intergenerational (16). Throughout this process, the hui reviewed the literature on land-based interventions, cultural practices and food sovereignty, and Hawaiian culture and values and discussed the findings with other cultural practitioners to aid in interpretation.

## Results

A total of 12 volunteers and five program leaders completed the survey and interview. Sociodemographic data are presented in Table 1. The participants were primarily female (*n* = 13, 77%), Native Hawaiian (*n* = 7, 41%), married or living with a partner (*n* = 7, 41%), had at least two additional family members in their household (*M* = 3.3, *SD* = 1.3), and had completed postgraduate education (*n* = 6, 35%). The next section includes exploratory quantitative and qualitative data assessing implementation strategies, including participation, satisfaction, and the receipt of products from the māla kalo. The qualitative data provide preliminary insights into the potential benefits of a māla kalo on the continent.

**Table 1:** Sociodemographic characteristics of the māla kalo volunteers and program leaders.

Demographic Data	*M* (SD)	*n* (%)
Age	42.4 (17.17)	
Sex		
Female		13 (76.5)
Male		4 (23.5)
Primary Ethnicity		
Native Hawaiian		7 (41.2)
White, non-Hispanic		4 (23.5)
Black		2 (11.8)
Japanese		2 (11.8)
Filipino		1 (5.9)
Korean		1 (5.9)
Marital status		
Single		5 (29.4)
Married/Living with a partner		7 (41.2)
Total adults and children in the household	3.25 (1.29)	
Education level		
High school graduate		2 (11.8)
Some college or Associate’s degree		2 (11.8)
4-year degree		2 (11.8)
Postgraduate education		6 (35.3)
Household income		
$10,000–$49,999		3 (17.7)
$50,000–$99,000		3 (17.6)
More than $100,000		5 (29.4)
Prefer not to answer		1 (5.9)

*N* = 17. Program leaders did not report marital status, household size, education level, or household income to maintain confidentiality. Primary ethnicity was self-reported from a list of 17 ethnicities, including a write-in option.

### Implementation strategies (participation, satisfaction, and products received)

Table 2 includes a joint display of the quantitative and qualitative data to evaluate participation in and satisfaction with the māla kalo, as well as the receipt of products grown in the māla kalo. The majority of the volunteers attended one or two workdays; however, the participants indicated that they wanted to participate more frequently. Among the program leaders, participation was assessed qualitatively, with one participant indicating that they traveled nearly 3 h to facilitate sessions, attending at least four or five sessions. All volunteers and program leaders indicated that they were satisfied with volunteering or supporting the māla kalo. However, they also identified areas for improvement, including increasing physical accessibility, addressing challenges in scheduling workdays, engaging the community, and expanding to other locations. Over half of the volunteers received a product grown in the māla kalo and shared the traditional foods prepared with other individuals. Unfortunately, due to limitations in data collection, we were unable to determine the reasons why products were not taken home (e.g., whether leaves were not ready for harvest or participants were not interested in taking products home).

**Table 2:** Joint display of the quantitative and qualitative data assessing implementation strategies.

Domain	Type of participant	Quantitative data	Qualitative data	Mixed-methods interpretation
Participation	Volunteer	41.2% (*n* = 7) participated one or two days 16.7% (*n* = 3) participated three or four days 11.8% (*n* = 2) participated more than seven days	One volunteer stated that they visited the māla kalo approximately 10 times, often bringing community members and attending outside of planned work days. Most volunteers who attended one or two days stated that they wished they were able to participate more often.	There was variation in participation, with most volunteers indicating they participated one to two days but wanting to participate more frequently.
Satisfaction with volunteering or supporting the māla kalo (program leaders)	Volunteer	91% (*n* = 11) were extremely satisfied 6% (*n* = 1) were somewhat satisfied	Volunteers reported challenges in scheduling and attending early morning workdays on weekends, but once they were at the māla kalo, they appreciated the sessions. They also wanted to share the experience with other people, were excited for the next growing season, and hoped to expand to other locations.	Volunteers and program leaders were highly satisfied but acknowledged challenges in running a volunteer-based program and expressed interest in exploring new ways to increase satisfaction and participation at the māla kalo.
	Program leaders	100% (*n* = 5) were extremely satisfied	Challenges included transportation, having workdays on weekends, sharing information beyond the members of the organization, and ensuring accessibility in activities for participants of all ages and abilities.	
Received products grown in the māla kalo	Volunteer	50% (*n* = 6) received at least one product from the māla kalo	Items received: Lau (leaves), prepared lau lau, beef luʻau, stew, and small plants. Volunteers reported sharing what they received with others.	Over 50% of volunteers received a product from the māla kalo and prepared it as a traditional meal to share with others.

### Benefits of a māla kalo on the continent

Preliminary benefits of having a māla kalo on the continent centered on developing and building pili or relationships. These relationships included connections to self, community, and land away from the islands; kaʻanalike ʻike (learning and sharing knowledge), specifically learning about and from kalo; and connection to culture through perpetuating cultural practices and protocols.

#### Connection to self, community, and land away from the islands

The participants shared the importance of the garden in establishing connections to identity, community, and land. For example, one participant described the realization that they need to take on their role as a kupuna (Elder) and share knowledge rather than contributing to the garden through physical labor. The participant shared,

“I was physically trying to act like I was back at 18, as everyone’s working, working really hard. But I turned around and I saw 2 ladies sitting, and I didn't sit at all. I saw what they were doing with the students as they were sorting the rocks into different sizes, and they were sitting there with the stick pointing. You know the kid would come up and show the Elder. The students had to say what the rock was [in the Hawaiian language] before they could put it in the pile… I thought, I should be taking my teaching skills and doing it that way because the building on the wall was taken care of by the young dads and the young moms and the teens… And I've been learning that, how it is important to be there, and Elders to be there and to be seen, as we are in Hawaiʻi.” [sic]

In this example, the garden created a space for the kupuna to realize their role as an Elder, the importance of Elders as teachers, and the need for Elders to be involved in activities, particularly when away from the islands. Similarly, the māla kalo became a place for the community across the lifespan to come together and share. For example, one participant said, “I loved seeing like the Elders and the youth together and seeing that intergenerational knowledge exchange happen. Kind of reminds me of when my grandparents and my uncles were in the garden with me when I was younger.” [sic] As a community living away from the islands, there are not many spaces for generations to come together and share, which can help facilitate community connections. Lastly, the garden created a place to connect with the community through the land. One participant shared:

“I think our community finds a sense of peace. Just being in a place and putting their hands in the soil and a reminder that no matter where you live, we are still people of the land, so you don't have to be in Hawaiʻi to know the soil.” [sic]

Being able to take care of the land together cultivated a space to connect with the community through the soil, creating a safe place, or puʻuhonua, for people of the land to gather while living away from the islands.

#### Ka’analike ‘ike (learning and sharing knowledge): learning about and learning from kalo

The participants shared that they not only learned about growing kalo but also learned from the plant itself. One participant shared:

“I understand what kalo can teach us, because it is the most amazing plant… and learning the story of Hāloa really personifies kalo…I feel different about it because I know the moʻolelo [story]… it takes on a whole different meaning. I don't want to use the word resilience, I want to use the word strength.”

This participant illustrated that by growing an origin story food away from the islands, the community can see strength and opportunity—that it is possible to continue to learn about Hawaiian culture and grow on the continent as a community.

#### Connection to culture: perpetuating cultural practices and protocols

The garden created a space for families to teach and learn cultural practices and protocols. The participants who brought their children often emphasized the importance of the garden in teaching cultural practices, language, and values—especially since their children, growing up away from the islands, have less access to these. For example, one parent shared:

“I want to instill these cultural protocols, values, experiences for my children growing up here on the continent so far away from our homeland…I need to teach them. And that’s another important piece of coming to the māla kalo or other cultural events like this it is that resurgence, that re-teaching of culture to the next generation that kind of been lost.” [sic]

Taken together, the māla kalo created more than a space to grow traditional foods for a displaced community. The māla kalo brought the community together to build relationships, learn, and connect with the land away from “home.”

## Discussion

Native Hawaiians are moving to the continent at an increasing rate, with more Native Hawaiians living on the continent than in Hawaiʻi. This exploratory evaluation of a land-based, culturally grounded program to grow kalo (a traditionally and spiritually important food) away from the islands shows preliminary promise. The volunteers indicated high levels of satisfaction, were interested in participating more often, and used the products grown in the garden. The preliminary findings indicate that the garden may cultivate connections to one’s own identity, each other, food, land, and culture while also offering a place to learn and share knowledge. These preliminary findings suggest that a community māla kalo may be more than a place to grow food; it can be a place to gather, share knowledge, and learn from plants.

Prior research has recommended the inclusion of a process evaluation, or examining implementation strategies, in the development of culturally grounded programs to ensure projects align with cultural practices, values, and expectations, as the lack of knowledge of cultural practices can be a significant barrier to implementation (6, 12). While our findings indicate high levels of satisfaction, we did not assess the implementation of cultural practices or engagement with cultural protocols and knowledge among the participants. As the first author holds a wealth of cultural knowledge as a kumu (teacher) of hula and is fluent in ʻŌlelo Hawaiʻi, we anticipated that the KALO HCC staff possessed sufficient cultural knowledge in the development and implementation of the māla kalo. However, future research may benefit from examining the implementation of cultural practices within this culturally grounded program.

We identified two other culturally grounded, land-based interventions developed for Native Hawaiians in Hawaiʻi: the Mini Ahupuaʻa for Lifestyle And Meaʻai (food) (MALAMA) program (29–31) and the Mauli Ola study at MAʻO Organic Farms. The benefits of the MALAMA program include improvements in diet quality (29), strengthened relationships to food and food sovereignty (21, 22), and a connection to the ʻāina as health, all of which align with our preliminary findings (31). While the MALAMA program shows promise in promoting health and wellbeing, it is home-based in Hawaiʻi and likely not transferable to other states with shorter growing seasons. Similarly, as a home-based program, the MALAMA program places less emphasis on gathering the community together, which the volunteers described as an important aspect of the garden. The Mauli Ola study, which evaluated a youth leadership training program focused on restoring relationships with the ʻāina to promote food sovereignty, education, health, and economic opportunities, suggests that food sovereignty and social justice programs may shape health trajectories among youth at risk for chronic disease (9). In addition, the methods used in the Mauli Ola study exemplify a CBPR approach and highlight the importance of reciprocal partnerships between the community and researchers to evaluate the potential benefits of culturally grounded, land-based interventions (9).

Similar to our findings, prior research evaluating land-based interventions with Indigenous communities—including subsistence farming and ceremonial practices—shows promise in promoting connections to one another, culture, land, and family, as well as highlighting the importance of upholding cultural practices, including learning from Elders (12). While community gardens have increased the consumption of fruits and vegetables in school settings (32), in Indigenous communities, they may hold additional benefits. By cultivating traditional and cultural foods, particularly those that are honored in origin stories (such as kalo), recognizing relationships to the land that feeds, and passing down intergenerational knowledge through growing, harvesting, and preparing food, it is possible to rehabilitate communities and cultures that were deeply impacted by colonization.

These findings are preliminary and have notable limitations. The small sample size, self-selection of volunteers, and cross-sectional study design limit any exploration of causality or generalizability. In addition, the use of descriptive statistics and exploratory thematic analysis provided preliminary data, and future research is necessary to expand on and validate these findings. We did not include culturally validated quantitative measures; therefore, the quantitative data may not reflect an Indigenous worldview. However, this study adds to the scant literature (33–35) recognizing the relevance and importance of supporting the displaced Native Hawaiian community through community-driven solutions.

As the Native Hawaiian community living outside of Hawaiʻi expands, these preliminary findings suggest that community gardens used to grow kalo may raise cultural visibility, create identity, and nourish the Hawaiian community by cultivating a deeply spiritual and revered traditional food. As other Native Hawaiian organizations on the continent have learned about the KALO HCC’s garden, they have requested assistance in developing their own gardens and learning how to grow kalo in a vastly different climate. This suggests two future directions: continued evaluation of the potential health benefits of a māla kalo on the continent and the development of resources to support other Native Hawaiian-serving organizations on the continent who are interested in implementing similar programs. Next steps include the following: (1) continued evaluation using culturally relevant measures such as ʻāina connectedness—a new measure developed in Hawaiʻi (31, 36) to assess measures of relational health—and Indigenous nourishment (37), a new measure developed by the American Indian and Alaska Native community; and (2) the development of the infrastructure to establish additional māla kalo, including creating manuals and providing technical assistance to support other communities.

## Data Availability

The raw data supporting the conclusions of this article will be made available by the authors, without undue reservation.
