# Supplementary material for: Growing kalo (taro) to promote culture and health in the Continental US
Source: Front Public Health. 2025 Oct 10;13:1689052. doi: 10.3389/fpubh.2025.1689052 (PMC12549674; doi:10.3389/fpubh.2025.1689052)
Supplement: Supplementary file 1 [file Data_Sheet_1.docx]

**Participant Interview Protocol**

*[Volunteering]*

First, tell me a little about volunteering at the māla kalo.

Which māla did you primarily support?

How many times did you work in the māla?

How did you hear about the māla?

What was the best part of working in the mala?

*[Community connections]*

Next, thinking about the Native Hawaiian and Pacific Islander community,

What connections have you made to the NHPI community since supporting the māla?

How can we grow interest for the māla among the NHPI community in Oregon?

How does the māla support your connection to culture and/or language?

What else could help you connect to NHPI culture and/or language?

What have you learned from participating in the mala?

What knowledge have you shared with others when participating in the mala?

*[Spiritual and Personal Connection]*

Next, we are interested in spiritual connections to kalo and growing kalo away from the islands, or on the mainland which, we also now call the continent. How has working in the mala supported your connections to kalo or the community on the continent?

What connections were you able to make with other community members since participating in the mala?

Did working in the mala improve your mental health? If yes, how? If no, why not?

*[Consumption and Preparation]*

Tell me about the last time you ate Hawaiian food. What did you eat? Tell me more about the experience.

Would you be interested in growing kalo in the future? Or is there anything else you’d like to learn about growing kalo?

*[If at University]*

What can we do to raise awareness of the māla on campus?

Considering this is a research project, what would help you to feel comfortable about sharing perspectives?

Mahalo for sharing. My last question is a general question. Is there anything else you’d like to share with us about this experience?

**[Ending]**

*Mahalo nui loa* (Thank you very much) for participating in this interview. We greatly appreciate your time and insight.

**Key Informant Interview Protocol**

*[Volunteering]*

First, tell me a little about volunteering at the māla kalo.

Which māla did you primarily support?

What was the best part of working with the mala?

*[Participation]*

Mahalo, the next questions are about volunteers, recruitment of volunteers, and raising awareness of this project.

What are challenges people face in helping with the mala?

What works well to get people to participate in the mala?

How could we raise awareness of the mala?

*[Community connections]*

Next, thinking about the Native Hawaiian and Pacific Islander community, what do people enjoy about working in the mala?

How does the mala offer place for community?

How does the mala support connections to culture among the community?

What else could help the community connect to NHPI culture?

What opportunities does the mala present to the community?

What knowledge is shared when participating in the mala?

*[Spiritual and Personal Connection]*

Next, we are interested in spiritual connections to kalo and growing kalo away from the islands, or on the mainland, which we also refer to as the continent. How has working in the mala supported your connections to kalo or your community on the continent?

What connections were you able to make with other community members since participating in the mala?

Did working in the mala improve your mental health? If yes, how? If no, why not?

*[Consumption and Preparation]*

Tell me about the last time you ate Hawaiian food. What did you eat? Tell me more about the experience.

*[If at University]*

How can we support the mala on campus?

How can we connect NHPI students on campus?

*[Research participation]*

Considering this is a research project, What procedures/protocols/governance needs to be in place to support research with the NHPI community?

Mahalo for sharing. My last question is a general question. Is there anything else you’d like to share with us about this experience?

**[Ending]**

*Mahalo nui loa* (Thank you very much) for participating in this interview. We greatly appreciate your time and insight.
