# Supplementary material for: Growing kalo (taro) to promote culture and health in the Continental US
Source: Front Public Health. 2025 Oct 10;13:1689052. doi: 10.3389/fpubh.2025.1689052 (PMC12549674; doi:10.3389/fpubh.2025.1689052)
Supplement: Supplementary file 2 [file Presentation_1.pdf]

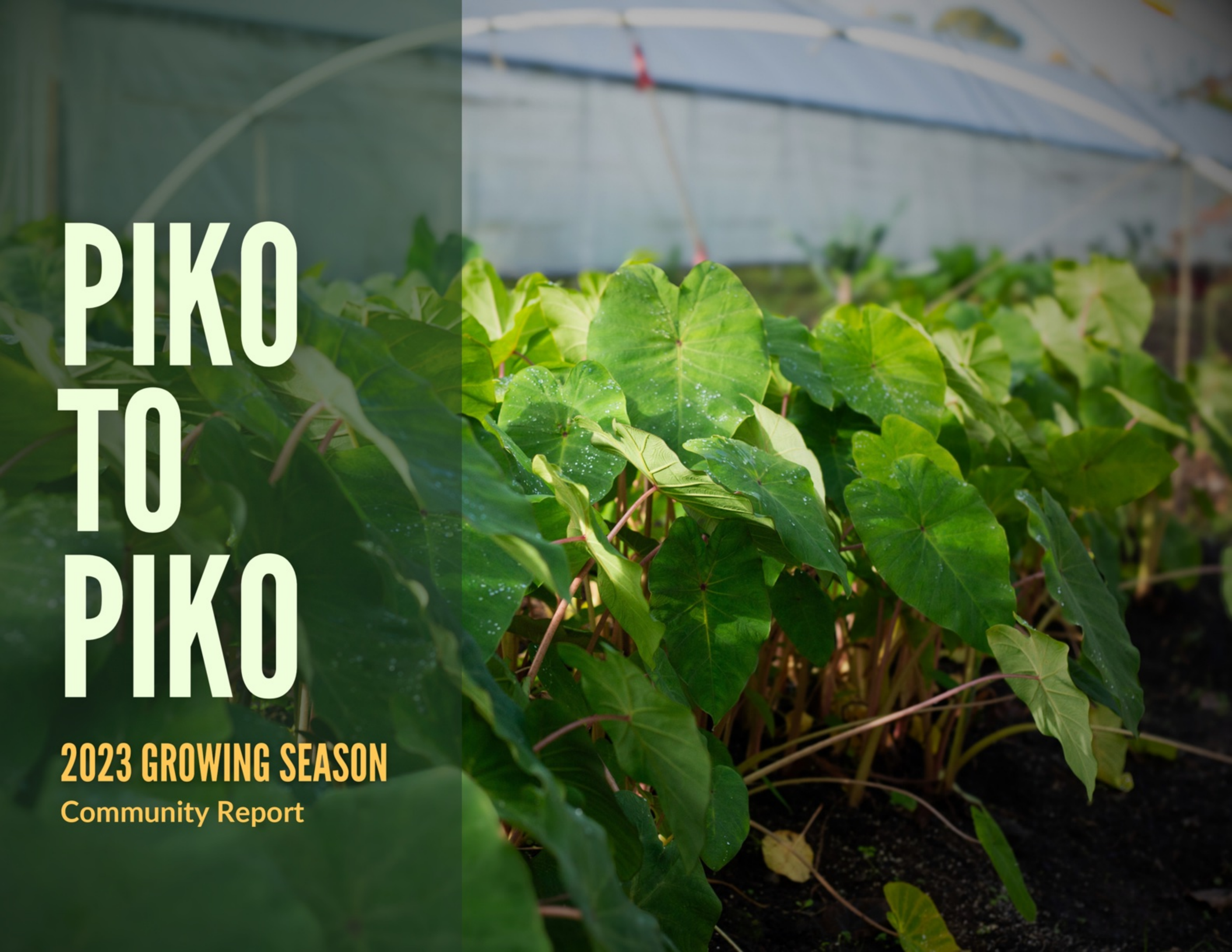

PIKO TO PIKO

2023 GROWING SEASON

Community Report

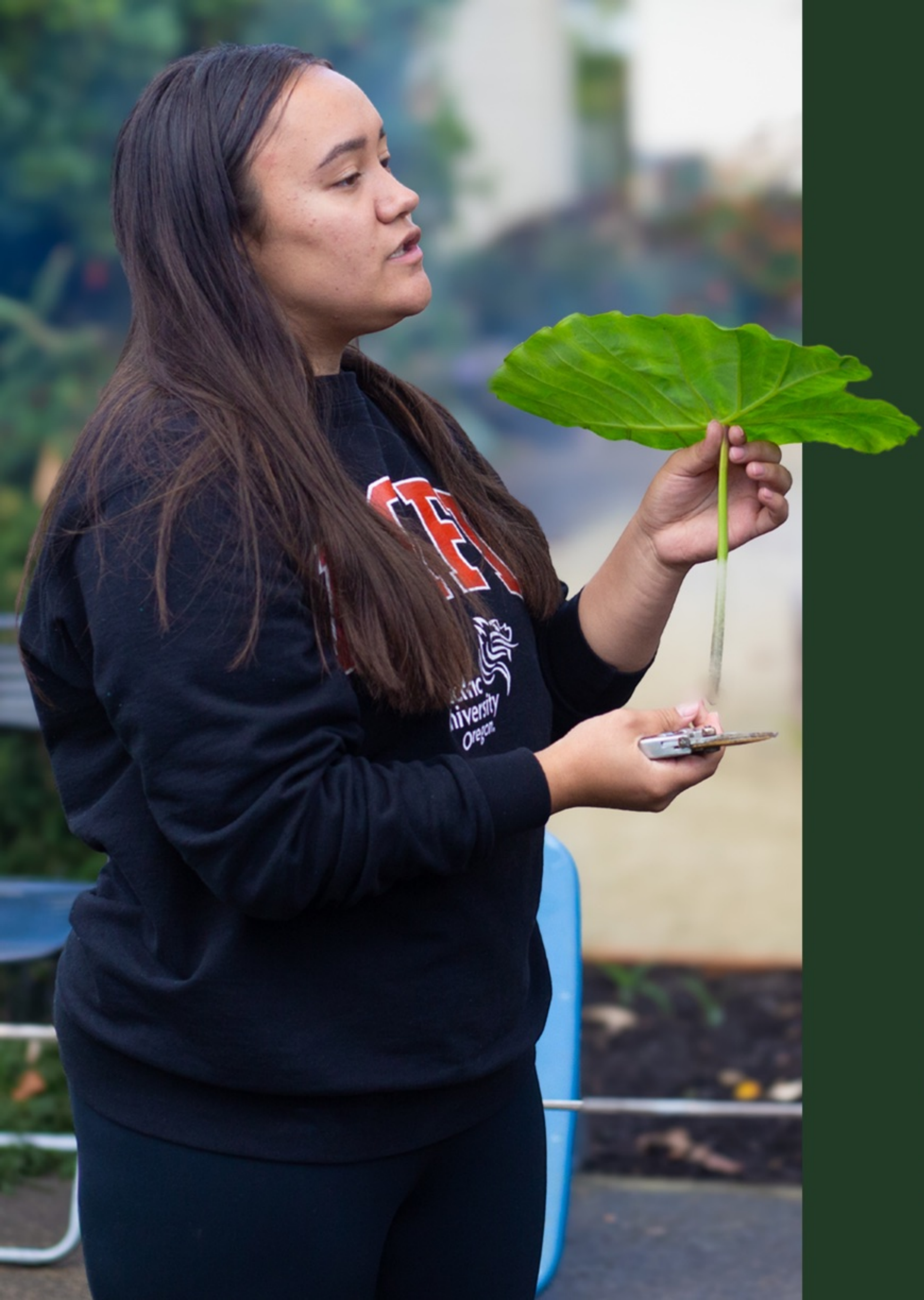

Table of Contents

Executive Summary 3
About Ka‘ Aha Lāhui O ‘Olekona Hawaiian Civic Club (KALO HCC) 4
- Mission 4
- Values 4
- Land Acknowledgement 5

Piko to Piko Hui 6
- Piko to Piko Hui 7-9
- Kuleana 6
- Piko to Piko Community Partners and Supporters 10

About Piko to Piko 12-13
- Māla Kalo Logic Model 14

Evaluation 15
Results 16
- Feedback about the Māla Kalo 16
- Benefits of the Māla Kalo 17-19

Conclusion 20
Moving Forward 21

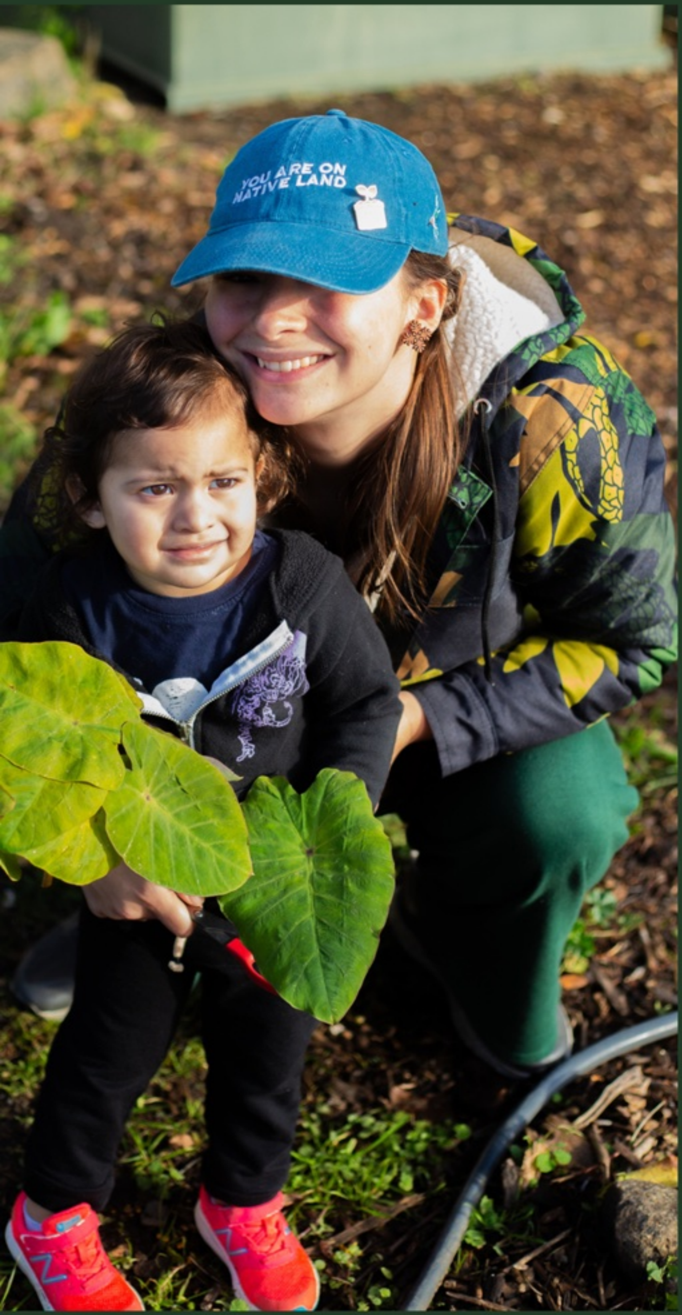

Executive Summary

Piko to Piko aims to understand the potential benefits of growing, preparing, and consuming kalo (taro) on the continent, away from Hawaiʻi, where many Ka ʻAha Lāhui O ʻOlekona Hawaiian Civic Club of Oregon and Southwest Washington (KALO HCC) members call home. Kalo is the origin of life for the Native Hawaiian people. Kalo is a deeply spiritual and sacred plant, and a staple of the Hawaiian diet. Kalo symbolizes the relationship between people, land, and ancestors, and growing this traditional food on the continent may not only increase access, but ground the community to the land in diaspora.

In 2021 KALO HCC established a māla kalo (garden to grow taro) at the Oregon Food Bank with the aim to bring the community together to grow this important traditional food. Since 2021, the māla kalo has grown both in size and in the community cultivating the garden. To care for the garden, community volunteers come together at least monthly and follow cultural protocols, learn Hawaiian values, practices, and language, share knowledge of growing kalo, maintain the garden, talkstory, work together in the dirt, and enjoy a meal together. Recognizing the potential health benefits of having a māla kalo on the continent, a hui of community members and researchers collected data from māla kalo volunteers during the 2023 growing season.

To understand the potential benefits, the hui invited māla kalo volunteers to complete an online survey followed by an interview from November - December 2023. We also invited five leaders of the garden to share their perspectives in a survey and interview. Data were then analyzed by the hui to evaluate if the māla kalo was feasible, or if it was something KALO HCC could successfully work with the community to offer, and what benefits the volunteers identified.

Overall, our findings were positive. Twelve volunteers and all five garden leaders participated in both the survey and interview. Participants greatly appreciated having the māla kalo and were satisfied with the activities at the māla kalo. Volunteers wanted to participate more often in future years. Participants shared the following benefits of the māla kalo: being together as a community in diaspora and connecting to land, learning about and learning from kalo, having a safe space or puʻuhonua, kaʻanalike ʻike (learning and sharing knowledge), perpetuating cultural practices and protocols, building a sense of identity and what it means to be Hawaiian on the continent, and physically connecting to the land away from "home." Potential benefits included developing relationships with the ʻāina, moʻo (lineage), ʻohana (family), and kaiaulu (community).

Based on these findings, the hui will collect data throughout the growing season in 2024 and beyond. By continuing to collect data, we hope to continue to understand how to support culture, nourishment, and thriving Native Hawaiian communities on the continent and how to develop the infrastructure to share these opportunities with other Native Hawaiian-serving organizations throughout the continent.

About

Ka ʻAha Lāhui O ʻOlekona Hawaiian Civic Club

Values – To advocate and elevate the voices of our Native Hawaiian and Pacific Islander communities through cultural practices and educational opportunities. To initiate change through education, collaboration and unity.

Mission – To actively participate in the promotion, perpetuation and practice of the Native Hawaiian culture and values.

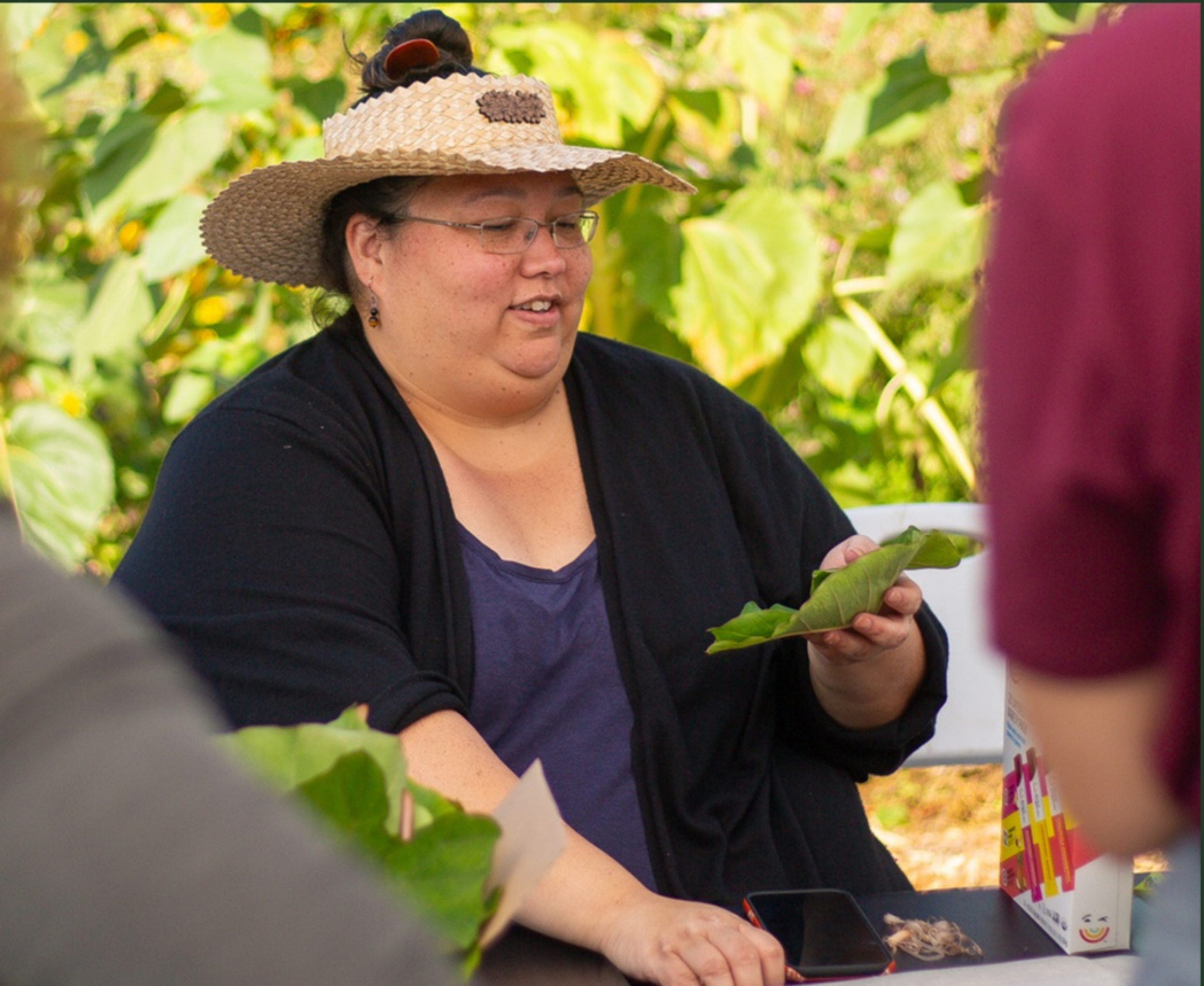

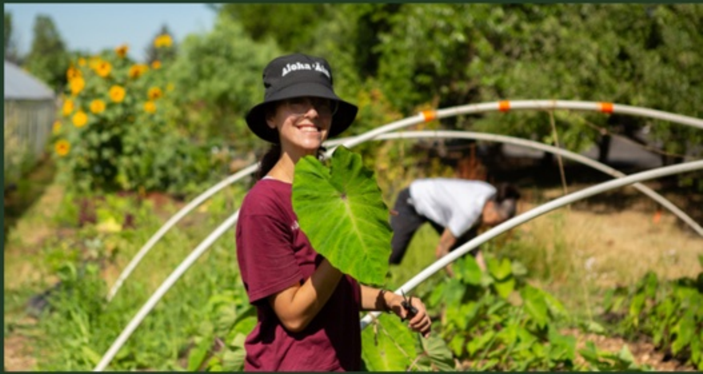

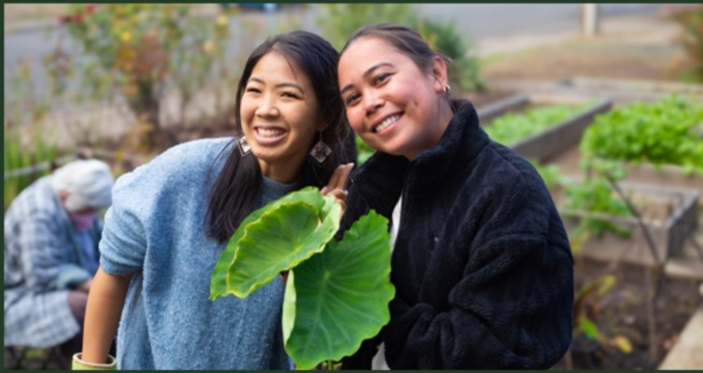

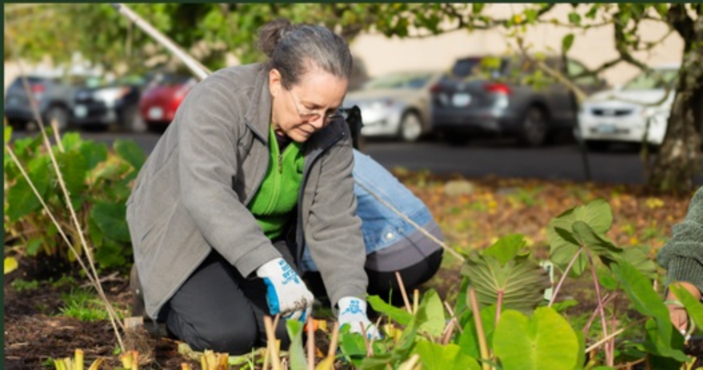

Land Acknowledgement

KALO HCC is located in what is now called Beaverton, Oregon. As Kanaka ʻŌiwi living on the continent, we would like to acknowledge the land that we currently occupy and stand on. We honor the traditions and culture of the Kalapuya tribe, the Confederated Tribes of Siletz Indians, the Confederated Tribes of Grand Ronde Cayuse, Umatilla, and Walla Walla, and many more Indigenous communities who are the original caretakers of the land.

It is important to recognize that while Pacific Islanders remain Indigenous in our own ways and in our own homelands, we are all simply visitors here. As a community-based organization, the mission and values of KALO HCC works to preserve Indigeneity within ourselves and with the people around us. It is an honor and privilege to share this space with our ʻohana as their legacies continue to live on.

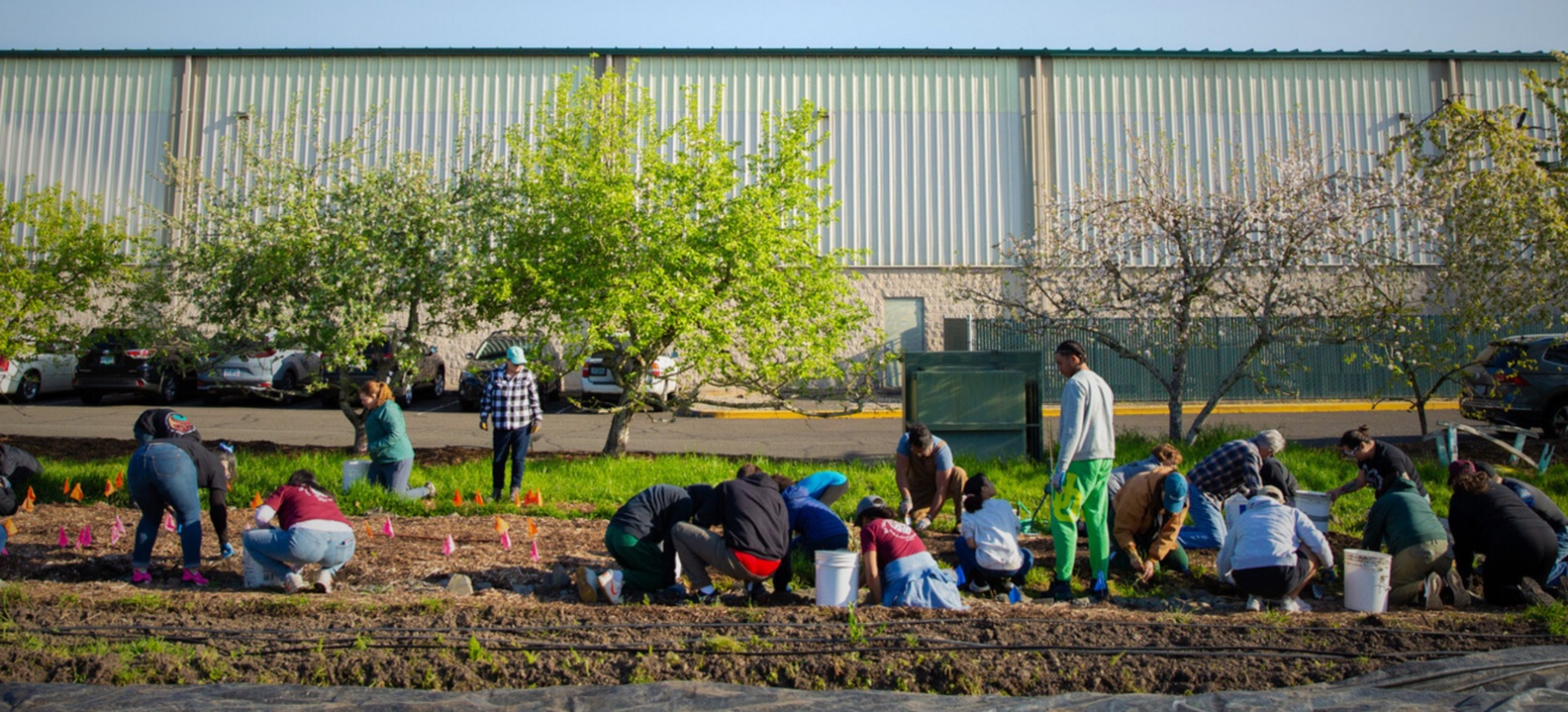

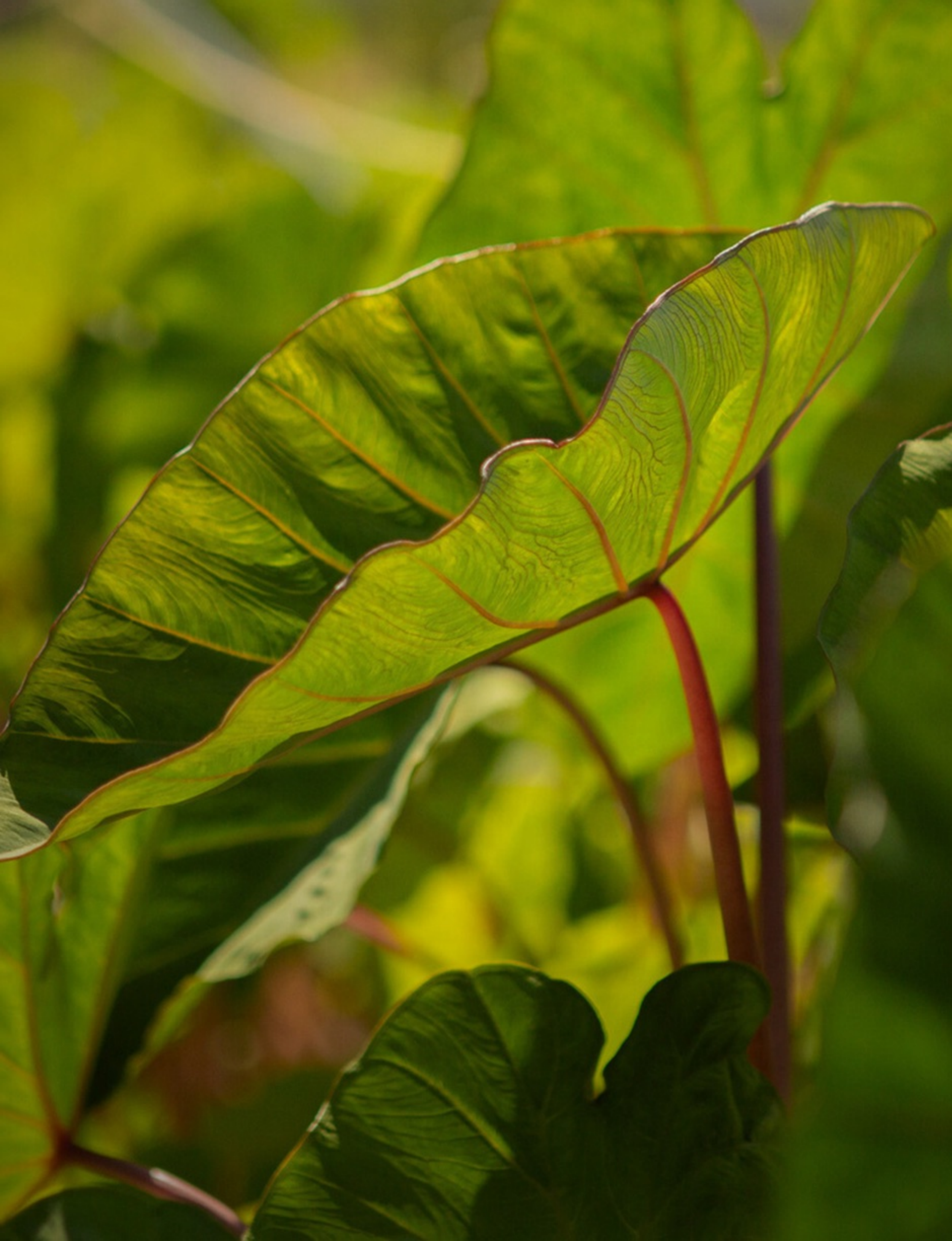

Piko to Piko Hui

How it started

The Piko to Piko hui was formed in 2023 to support and evaluate the māla kalo as part of the KALO HCC's Health and Public Policy committee. The hui supporting Piko to Piko includes community members, KALO HCC leadership, interdisciplinary researchers, and undergraduate and graduate students, all of whom are Asian American or Pacific Islander or have connections to Hawaiʻi. All members of the hui dig together at the garden, engage in cultural traditions, and share their manaʻo (insight) to uplift the lāhui (tribe). The foundation of Piko to Piko is the shared kuleana and connections to one another and the community.

Kuleana

In ʻŌlelo Hawaiʻi (Hawaiian language), the word kuleana translates to responsibility, privilege, and concern, and is often used to explain one's sacred duty. Kuleana is deeply tied to the personal relationship Native Hawaiians have with the ʻāina and culture. The Piko to Piko hui shares our kuleana to highlight the perspectives and experiences within this project, as well as what drives us to help achieve the goals of Piko to Piko.

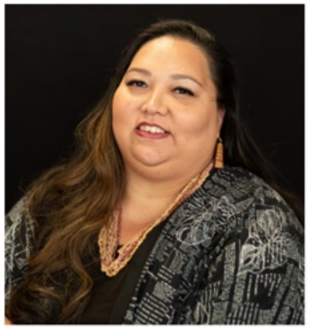

Leialoha Ka'ula

Aloha kākou! ʻO wau no ʻo Leialoha Ka'ula a he kama au ʻo ka pae ʻāina ʻoi kelakela ʻo Hawaiʻi. Born on the island of Oʻahu, raised on Moku O Keawe, and now humbly appreciate the lands of the Atfalati tribes in hopes to uplift their stories and community. As a hula practitioner, cultural practices and the stories of our ancestors is the foundation of all that we do. These practices and protocols that have been instilled since a young age continues to guide me each and every day. As a proud graduate of Ke Kula ʻO Nāwahīokalaniʻōpuʻu, ʻŌlelo Hawaiʻi surrounds all that we do and has allowed for me to carry what has been taught to me here in the Pacific Northwest. In 1918, Prince Jonah Kuhio Kalanianaʻole founded the Honolulu Hawaiian Civic Club with a vision to engage our lāhui in the need to be civically involved in matters that impact our community. Today, the Association of Hawaiian Civic Clubs is home to over 60 chartered clubs that stretches across Hawaiʻi and Moku Honu (Turtle Island). Ka ʻAha Lahui O ʻOlekona or KALO is one of those proud organizations with a mission and a vision to carry out the work of our Prince. Living in diaspora is a challenge that so many of us are faced with and don't have the tools to navigate when it comes to how we can make an impact here in the Pacific Northwest. Our Aliʻi were world travelers - navigators! Here at KALO, my goal is to establish, grow, and maintain relationships within our communities here on the continent and in Hawaiʻi to insure our lāhui, ʻohana from Hawaiʻi, and our Pacific Island communities feel uplifted, seen, and heard. WE are the IMPACT and the CHANGE for we are ALOHA. - Maikaʻi ke kalo i ka ʻohā!

Nicole Lee Kamakahiolani Ellison

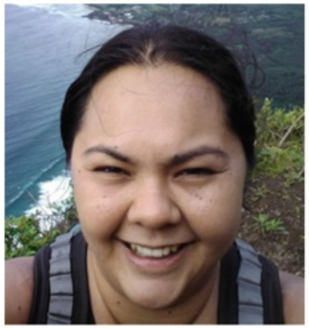

Howzit! Wai'anae girl at heart here living in the Pacific Northwest. I am blessed with being able to serve our community as the KALO Director 4. I help advise Piko 2 Piko through a leadership role that is driven by community. I am excited about changing the view on data and learning more on how to serve my community. Generally, I approach research and evaluation as how I would be if my own Tutu was part of the community involved. How would she want to be included and how can I make her feel respected and valued? My actions are influenced by Native Hawaiian values, deep ties to caring for the land as a sibling, and trying to figure out how to navigate being kanaka while living in the diaspora.

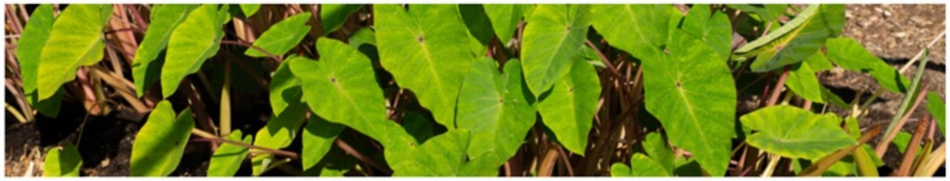

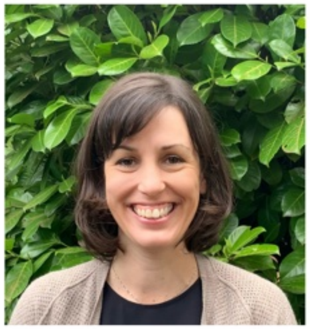

Alexandra Jackson

I am still learning my kuleana; in the world and related to this project. Currently, I see my kuleana as: to honor the knowledge shared by mentors with me and to do my best in sharing the stories of the communities I have the privilege to be a part of. I am a forever student - learning from incredible researchers, community leaders and organizers, and community and cultural protectors. Through these experiences, I am slowly putting together what I know and need to continue to learn. In this project, I see my kuleana as to work with the community to find ways to sustain Piko to Piko and tell our story of how a community māla kalo is central to cultural perpetuation and health promotion on the continent.

Jonathan Cruz

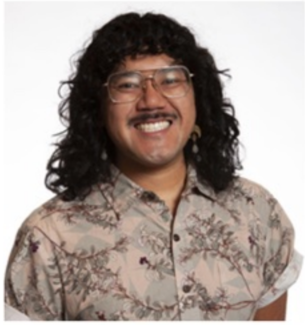

My kuleana is to our people's history of resistance from imperialism and colonialism by centering our people's way of knowing and relating to land. Indigenous resistance is critical to ecosystems, our health and wellness, and the survival of the Earth. From Standing Rock to Maunakea, we keep our culture alive and pass our wisdom to our keiki through working with the land, wherever we are. I'm grateful to be a part of this project in making knowledge, defining health for us and by us, and bringing our community into our process.

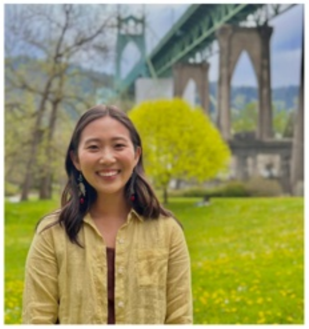

Sooyoun Park

As someone who was nourished by my grandmother's and mother's cooking, one of the first things I learned from my family was that food is the language of love and togetherness, and can carry stories, holding deep memories, across generations, time, and space. My identity as the daughter of Korean immigrants continues to shape and guide my work with diverse communities to create a future in which all people are healthy and whole, and accepted. As a prevention science student, I explore preventative programs and interventions that acknowledge the complex, intersectional and systemic issues which impact health and well-being. My work is grounded in the deep belief that a society which uplifts and honors Indigenous life and ways will also be one that uplifts the lives of all people, particularly those who have been oppressed and marginalized since the inception of this country. I am deeply grateful to the communities she has the privilege of working with, who continue to guide her in advancing health equity. Outside of my studies, I try to spend the rest of my time doing things that bring me joy with the people I love, some of which include yoga, gardening, and of course– cooking and eating together.

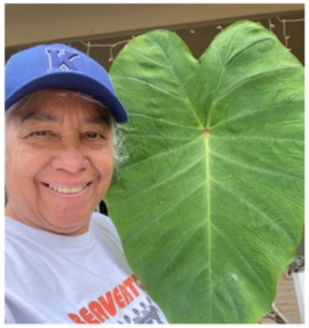

Louise Wilmes

My name is Louise Kalahikiola Wilmes. I am a member of the Otoe-Missouri Tribe of Oklahoma and Native Hawaiian. I was born in Honolulu Hawaii and grew up in Kalihi surrounded by Native Hawaiian family and neighbors. Our playground was the Bishop Museum where Hawaiian artifacts and "spirits" reside. I attended Kawaiahao Church and graduated from The Kamehameha Schools. Then I attended and graduated with a Bachelor of Arts in Elementary Education from Pacific University '75 in Forest Grove, Oregon and then earned a Masters of Education in Education Administration from George Fox University '00 in Newberg, Oregon. I taught grades K-6 in Beaverton, Oregon for 41 years. I married an Oregonian and raised my family in Oregon. My life has been defined by these 2 different "worldviews"; immersed in Hawaiian culture and then trained by the Western view in education and teaching.

Three years ago, I learned the Story of Haloa: Creation of our kalo ancestor. I began a transformation of thinking about the importance of finding my "piko" while living on the Continent. I began to grow kalo in my backyard. This year, I started a very simple journal about my experience of learning how to plant, tend to, harvest, and cook the kalo. This very informal study has inspired me to re-think how to plant Native Hawaiian food in the future and share it with our Hawaiian community in Oregon.
I have so much respect for the kalo and its determination to survive and provide for us. This photo is me with the biggest kalo leaf of the week.

Mandi Suzuki

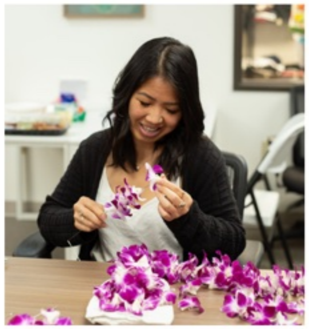

One of the many values that is deeply embedded in the Hawaiian culture is mālama - not only caring for one another, but also the land, waters and air that nourishes and sustains us. My kuleana, and my hope, for engaging in this Piko to Piko research is to cultivate and nurture the value of mālama through cultural education, awareness and community practices of growing kalo on the U.S. Continent. Although I do not identify as Native Hawaiian, my roots and upbringing in Hawai'i fostered a sense of responsibility to preserving Hawaiian language, traditions and culture, especially amongst a growing diaspora of Hawai'i natives to the U.S. Continent. My privilege of attending higher education is also embedded in my kuleana in uplifting, advocating and empowering NHPI voices through community based participatory research.

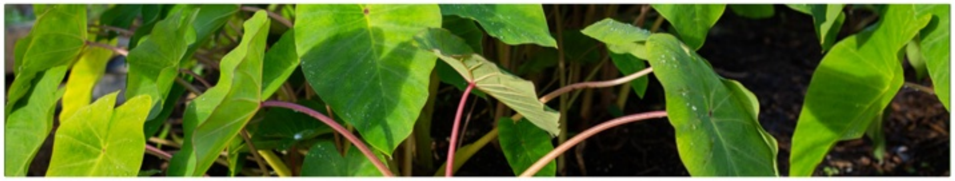

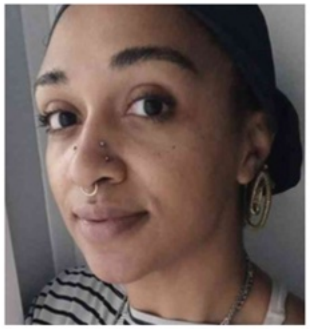

Constance "CJ" James

I am the Mala Support lead. My kuleana within that position is to maintain the mala and welcome community members into the space with aloha. As a Black person with no (known) ties to Hawaii, a large part of my kuleana is learning as much as I can about Hawaiian culture, language, food, health, traditions, and diaspora. All of this is relevant to my work. I spend a lot of time with the kalo. Getting to know kalo is my job, but it has become a personal experience. I feel dedicated to the well-being of the mala and everything we choose to plant in it. As a Black person working for a Hawaiian non-profit, I naturally feel the need to network with other BIPOC organizations. I have learned that kalo has a place in my ancestral Nigerian diet. Learning about this has inspired me to learn more about migration, trades, routes, and cultural exchanges throughout history. This is, indeed, a Hawaiian Civic Club. The focus should always be Hawaii *and* networking or learning from other groups (Indigenous, African, South American, etc) can only strengthen our collective land-tending knowledge. My name is Constance. It means "loyal, true" I feel that the health of the mala is attributed to my consistency in my visits to it. My kuleana (as it pertains to P2P) is to keep records about mala produce production, mala health, and volunteer attendance. It is also to connect with the volunteers and help the executive director facilitate educational moments at the mala. More than this, I am to be a friend of the mala as kalo is very pleased when it has consistent attention.

Marla Alohilani Tam-Hoy Barhoum

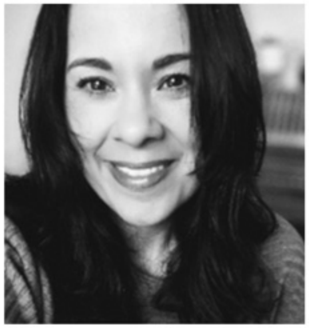

My name is Marla 'Alohilani (Brightness of the Heavens) Tam-Hoy Barhoum. I am proud to be Kanaka Maoli (Native Hawaiian), born and raised on the continent in Seattle, WA. I walk in two worlds as my Dad is Native Hawaiian and my Mom is of European descent. I am hapa. I live in gratitude. My parents instilled Hawaiian cultural values in our home. They connected me to my Hawaiian-ness. My Dad sat on the board of the Hawaiian Civic Club, he co-founded Kīkaha o Ke Kai (to glide swiftly across the water) Outrigger Canoe Club and Lōkahi (unity), a non-profit to perpetuate our culture here on the continent. I grew up listening to Hawaiian music; he played the ukulele, guitar, and bass and sang to us. This is how I learned our language, through mele (song). My Dad's aloha spirit and cultural activism was my inspiration to follow in his footsteps. To be connected, aware, and kōkua (help) when there was a need in our community. It is now my kuleana (my responsibility) to protect and nurture the health of my community, while upholding our cultural values and belief systems.
My career path has always centered on serving others, especially those that are both underrepresented and underserved. I spent over 25 years in education (many of those years spent working with local Coast Salish Tribes), social work, and curriculum development.

I am passionate about education and health research and consider myself a life-long learner. I currently work on two Native Hawaiian and Pacific Islander health studies through Washington State University's Institute for Research and Education to Advance Community Health (IREACH). The first, Healthy Hearts Among Pacific Islanders, is an educational-based study. Its curriculum was created by and for Native Hawaiians to manage pre-diabetes, diabetes, and/or hypertension and is the first online research study for our people. In this project, I get to weave my passion for health education and health research.

The other, Moana: Alternative Surveillance of COVID-19 in a Unique Population, is a respiratory illness study. Recruitment ended in October, and I am excited about the opportunity to do statistical analysis, disseminate results to the community, and collaborate on manuscripts for peer-reviewed health sciences journals.

The fall harvest and time of makahiki incorporates ceremony, community, and sharing along with feasting on healthy foods. In the spring, seeds are planted, which will flourish later; it is symbolic. I feel my ancestors gently pushing me forward, on this journey. Native Hawaiians are understudied, and more data is needed to help the health of our people. I want to participate in research using the Indigenous framework of "by us, for us." I commit to relational accountability through trust, respect, and reciprocal relationships. I have three keiki, the oldest lives in Los Angeles and we live in Mukilteo, WA. I have two teen daughters in high school that love to play basketball, so we watch a lot of games and travel together for AAU tournaments. I enjoy traveling, reading, photography, hikes, spending time at the beach, yoga, kickboxing and hip-hop dance classes and taking walks (and cuddling) with our dog, Chewie.

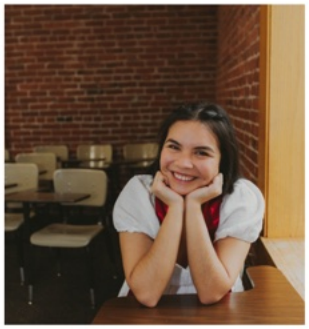

Kawehilani Wong

My name is Kawehilani ('Kawehi' meaning adornment, and 'lani' meaning heaven/heavenly) and I'm the youngest daughter of three children. My dad is Native Hawaiian and was born and raised in Kāneʻohe and my mom is Ecuadorian and was born and raised in Guayaquil, then later immigrated to the U.S.. From a young age my parents worked hard to ensure that my siblings and I were constantly surrounded by our own culture and traditional experiences. I grew up eating many traditional foods, listening to Native Hawaiian music, dancing hula, and learning to speak and understand both ʻŌlelo Hawaiʻi and Spanish. Living on the mainland made some of these cultural practices harder to continue as time moved on, but during my time in undergrad I was reintroduced to the Kumulipo (Hawaiian Creation Chant) and it became a driving force in the further development of my cultural understanding allowing me to reconnect with who I am and where I come from. Through my educational background I developed a passion for maternal and children's health, addressing food insecurity among the Native Hawaiian community, and finding ways to adapt existing care to better fit the experiences and values Native Hawaiians carry with them everyday. Knowing this, my k-uleana is to my family, my work, my people, and to future generations. To always learn from them, grow with them, lend support when I can, and to make them proud. In 2025, I will graduate with my Master's in Public Health in Health Promotion. I'm lucky to have the privilege to receive the education and experience I have so far and in return I plan to be a steady vessel for knowledge and continued perpetuation of Native Hawaiian culture. It is my hope that I can be an empowering presence allowing voices from marginalized backgrounds the chance to reclaim their identity.

Kacyn Ideue

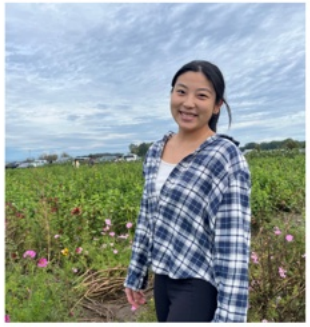

Aloha! My name is Kacyn Ideue, and I'm currently an undergraduate student at Pacific University studying both biology and public health. As a future health practitioner, I see my kuleana as being both a life-long learner as well as an advocate for Native Hawaiian and Pacific Islander health and culture. My hope is to grow in knowledge so that I can be better equipped to support the Native Hawaiian patients living in diaspora with the necessary resources and positive outcomes.

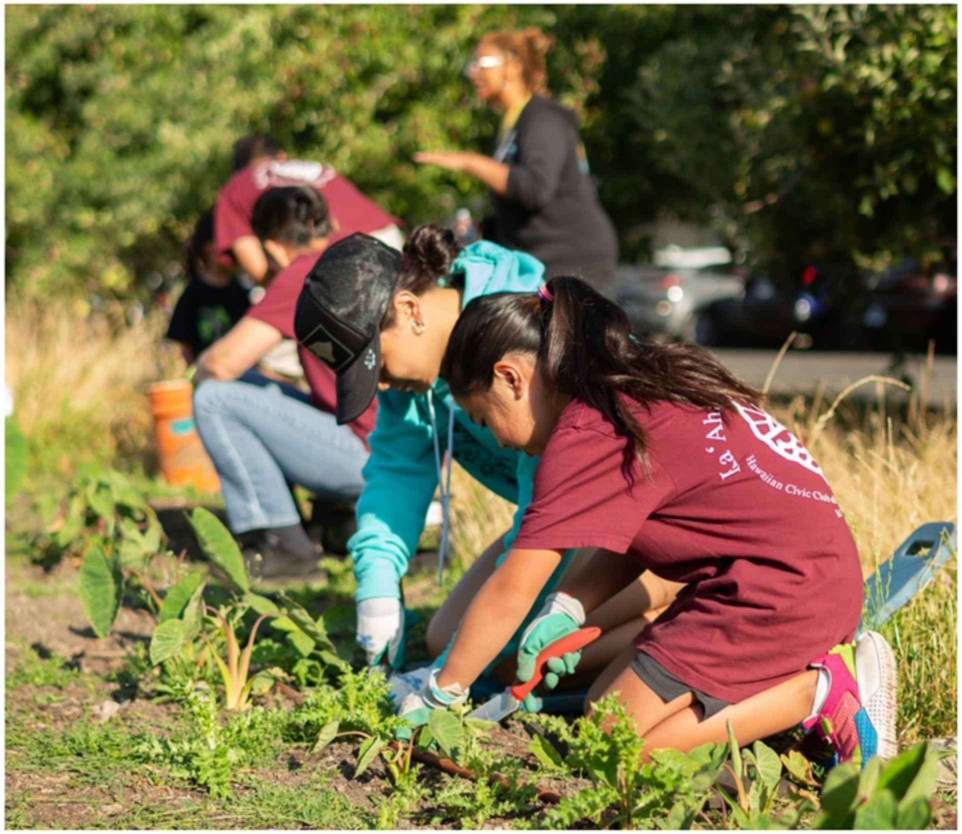

Community Partners & Supporters

- The Oregon Food Bank
- Papa Ola Lōkahi
- Oregon Metro
- Oregon Community Foundation
- Oregon Pacific Islander Coalition
- Pacific University
- Kindness Farm

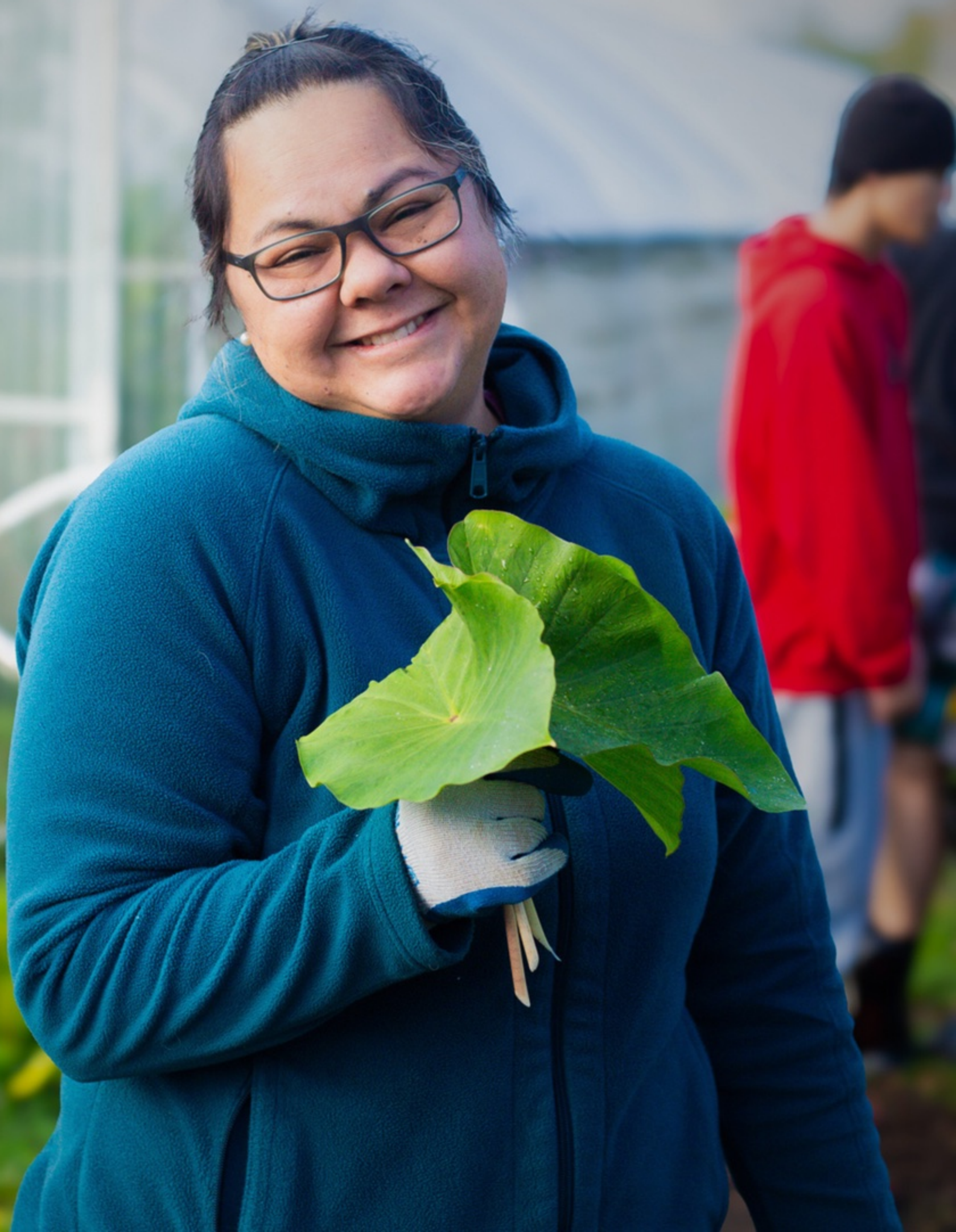

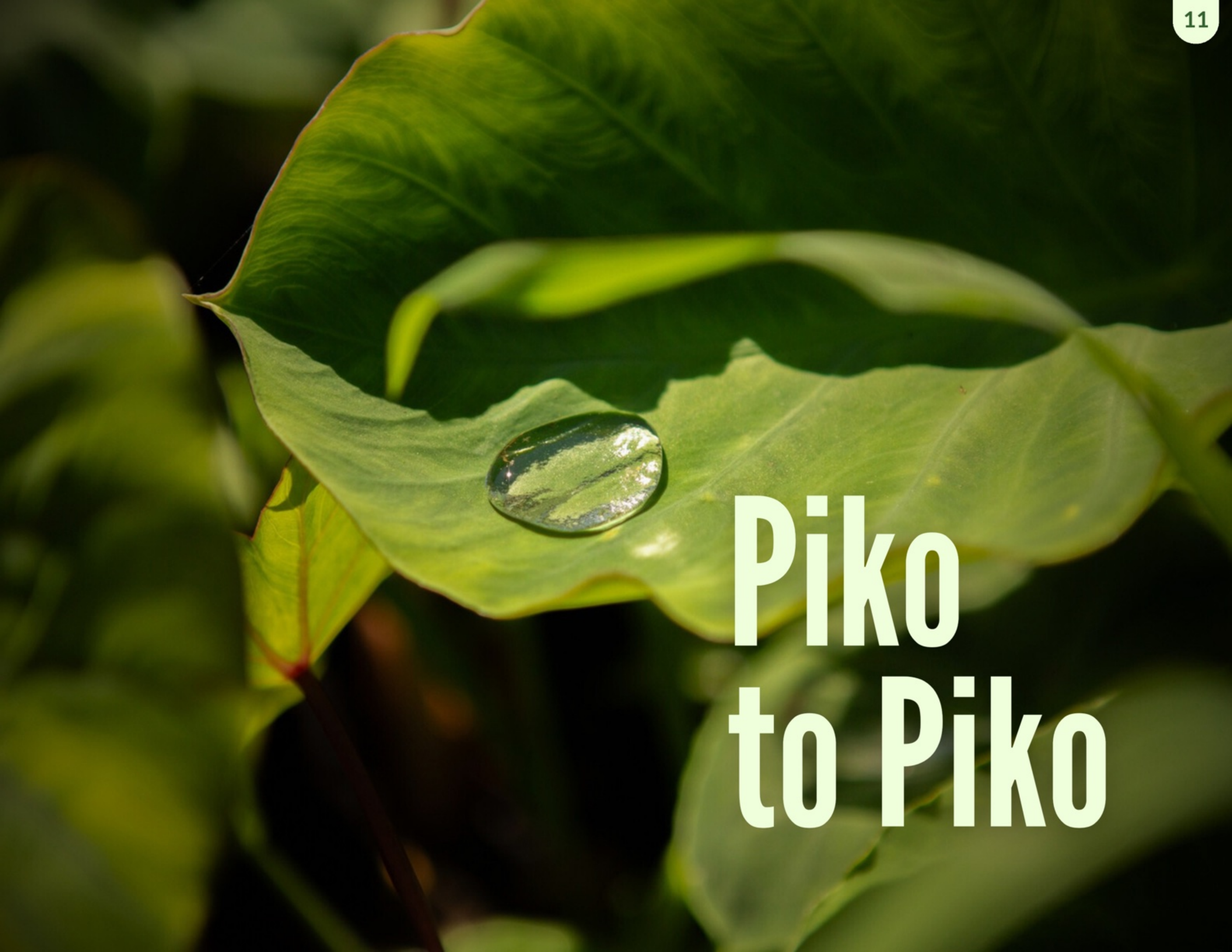

Piko to Piko

About Piko to Piko

Piko is defined as our umbilical cord. In Hawaiian culture, there are three piko - linking the past, present, and future; connecting to our ancestors, our ʻohana, and the future generations we create and establish. We call the māla kalo "Piko to Piko" as our work at the māla kalo links our past, present, and future - including our connections to one another in diaspora and our past connection to the ʻāina (land).

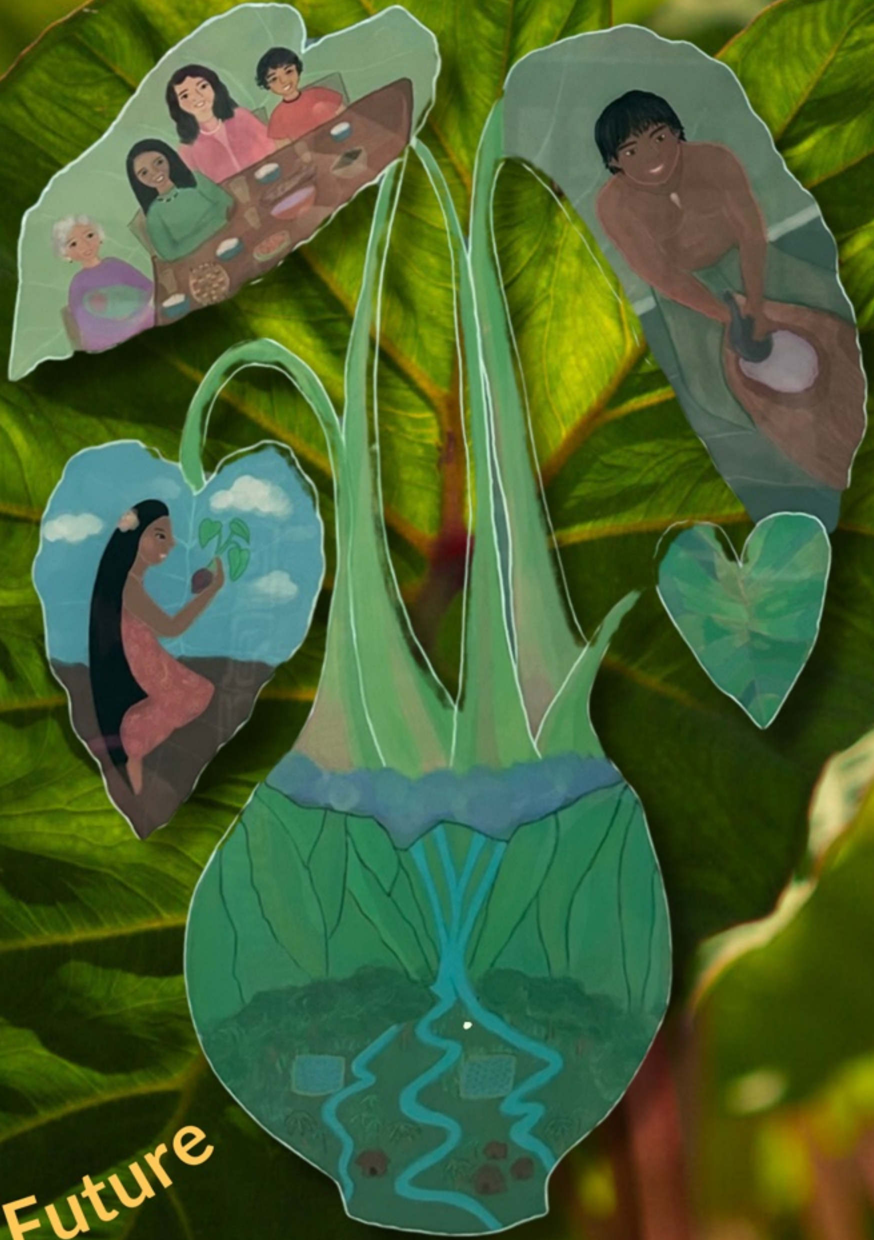

Connecting the Past, Present, & Future

About Piko to Piko

In this section we share why the māla kalo is significant to the Native Hawaiian community on the continent including the kumulipo (Hawaiian creation chant), beliefs of Prince Kūhiō, and importance of connecting to land or ʻāina.

1 The Kumulipo

Hawaiian Creation Chant

The kumulipo (Hawaiian creation chant) describes the significance of kalo. According to the kumulipo, Hāloanakalaukapalili was stillborn and buried in the ʻāina by Papahānaumokuākea at the request of Hoʻohōkūkalani. Born from this child in the ʻāina is kalo, a plant that nourished the second born son, Hāloa who all Native Hawaiians trace their lineage to. Connecting the community,ʻāina, and kalo; the piko of the Native Hawaiian community and culture.

2 Prince Jonah Kūhiō Kalanianaʻole

Founder of the Association of Hawaiian Civic Clubs

Prince Jonah Kūhiō Kalanianaʻole, the founder of the Association of Hawaiian Civic Clubs was a strong advocate for the connection to land. Prince Kūhiō stated "After extensive investigation and survey on the part of various organizations organized to rehabilitate the Hawaiian race, it was found that the only method in which to rehabilitate the race was to place them back upon the soil," creating the Hawaiian Homes Commission Act that was intended to return Hawaiians to the land. Piko to Piko builds on this belief - to rehabilitate and nourish Native Hawaiians living on the continent, it is crucial to cultivate connections to the land.

3 Connection to land or ʻāina

Cultivating a connection with land can also be understood in definingʻāina. ʻĀina is often translated as "land," however, ʻāina can be translated as "the land that feeds," highlighting the reciprocal relationship between land and people who mālama (care) for the land. When defining ʻāina as "the land that feeds," that indicates the land provides for those who reside on it and in return, we need to care for and nourish it in return.

The māla kalo creates a piko for the Native Hawaiian community to perpetuate cultural practices on the continent, building identity, belonging, and a puʻuhonuna while cultivating language away from "home," the piko we came from. At weekly and monthly workdays, volunteers follow established protocols, learn Hawaiian values, practices, and language, share their knowledge of growing kalo, talkstory, work together in the dirt, and enjoy a meal together. Most workdays, volunteers are able to take lau (leaves, used in food preparation) and small plants home to establish their own gardens.

Piko to Piko and how we understand the potential benefits of the program are informed by the Ahupuaʻa model developed by Papa Ola Lōkahi and the Indigenous Connectedness Framework, developed by Jessica Ullrich. The Ahupuaʻa model centers cultural strengths and the importance of ʻohana, traditional knowledge, interdependence, and respect for the land. The Indigenous Connectedness Framework describes four domains of connections: environmental, family, community, and intergenerational that contribute to collective health and wellbeing.

For more information, visit:
https://www.papaolalokahi.org/program/ahupuaa-model and
https://doi.org/10.1177/1177180119828114

Improved mental, physical, & spiritual health

Identity development on the continent

Thriving community on the continent

Connection to ʻāina

Intergenerational transmission of cultural practices

Increased access to traditional foods and growing kalo

Increased knowledge of cultural practices

About Piko to Piko

Māla Kalo Logic Model

To help describe Piko to Piko, the hui developed a culturally-grounded logic model (included below). At the heart of this model is the kalo plant itself, which our work grows from. In dark brown, which aligns with the dirt that cultivates kalo is what we need for the program itself. In lighter brown, we include our outputs that can help to identify if we are able to offer the program as we intended. The blue is the sky, or what benefits we hope to see in the community with the lightest blue being the most distant aspirations.

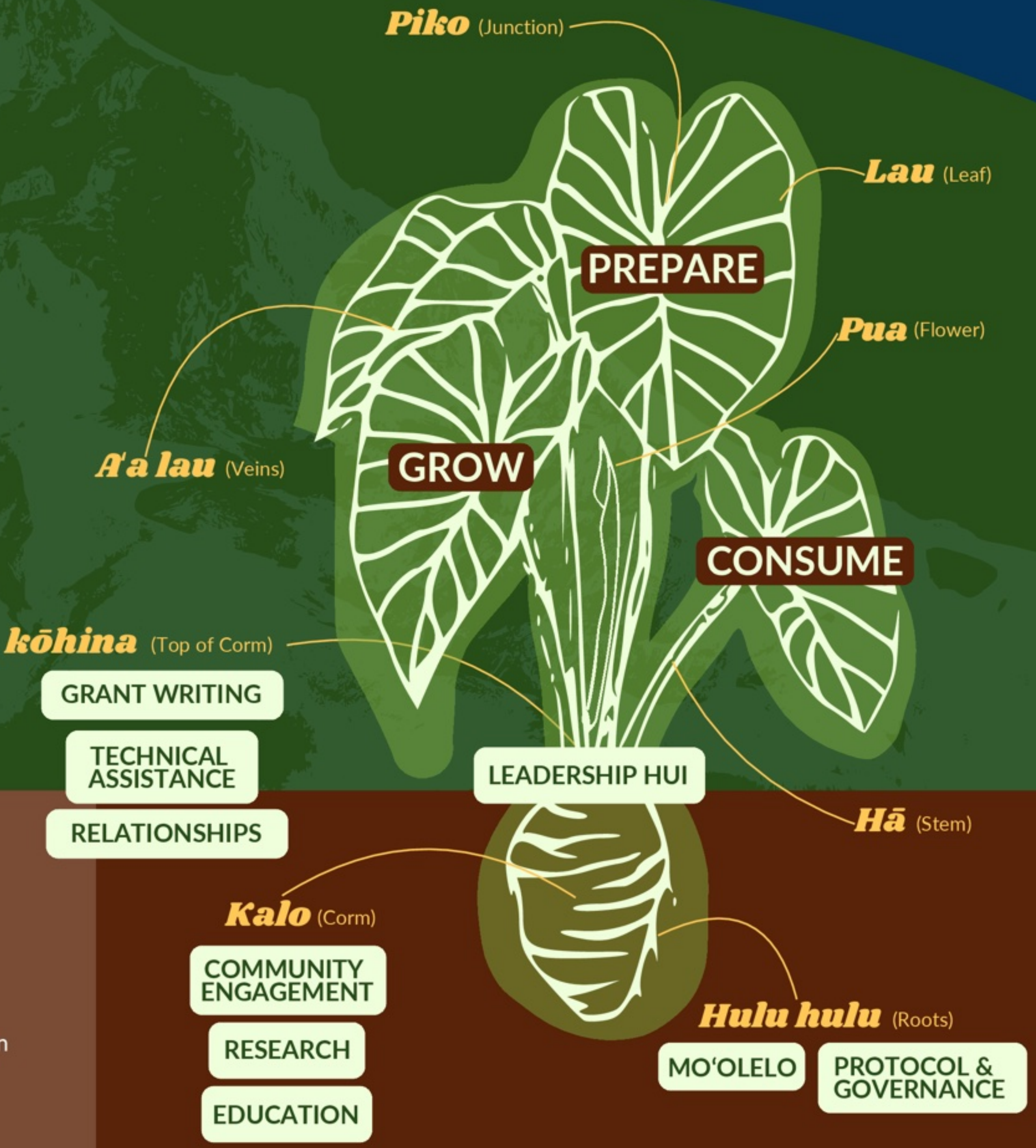

Activities

Host bi-monthly community work days including cultural protocols & practices, gardening, talk story, sharing food, & products grown in the māla kalo

Outputs

- Community participation
- Satisfaction
- Sharing products grown in the māla (food & plants)

Evaluation

We invited five garden leaders and all volunteers 18 and older who participated at the māla kalo during the 2023 growing season to complete a survey and interview to:

1. Identify ways to improve Piko to Piko or the māla kalo program.
2. explore potential health benefits of this program

Volunteers were contacted after the last māla kalo harvesting day to take part in a 15-minute online survey. Māla kalo leaders were asked to complete a 5-minute online survey. After completing the survey, participants were invited to complete an online interview to learn more about their experiences at the māla kalo. Interviews took 30 minutes to 1 hour to complete. Survey and interview questions are available on request. Participants were compensated $30.

The evaluation of Piko to Piko was guided by the eight phases of Māʻawe Pono, a research method developed by Dr. Kū Kahakalau and Dr. Manulani Meyer. In ʻŌlelo Hawaiʻi, māʻawe means a narrow path or trail, and pono means what is good, right, and just. The name māʻawe pono was chosen because, like the ancient trails that helped our ancestors travel through tough terrain, this method provides a path for Hawaiian researchers to tackle current challenges, restore fairness, and bring about justice. Through this approach, we follow the teachings of our ancestors, honor the past (ka wā imua), and base our what we learn in Hawaiian tradition.

All results were shared with the community and feedback was incorporated prior to publishing findings with a wider audience. During the community review process, we let participants whose quotes were included in this report either choose to use their name or a pseudonym of their choosing to recognize their contributions to this project.

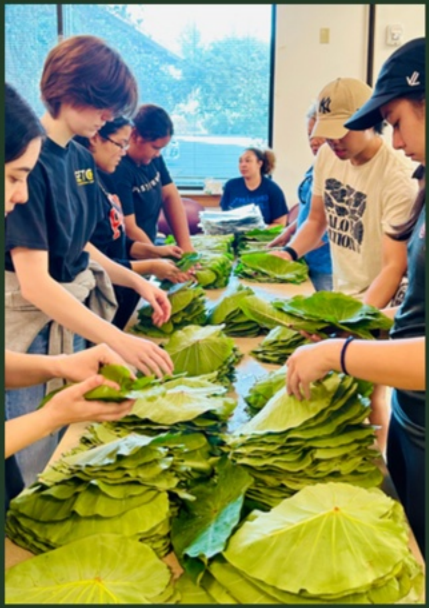

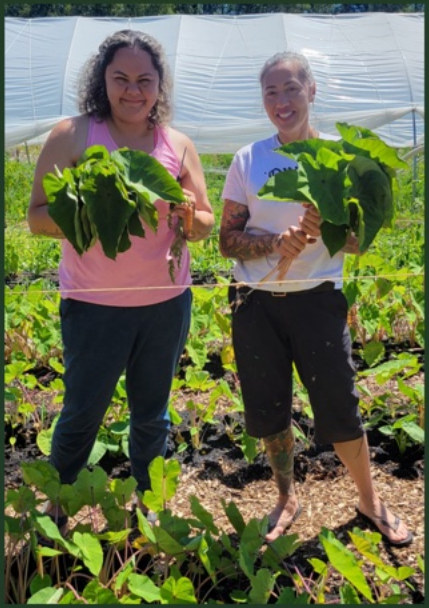

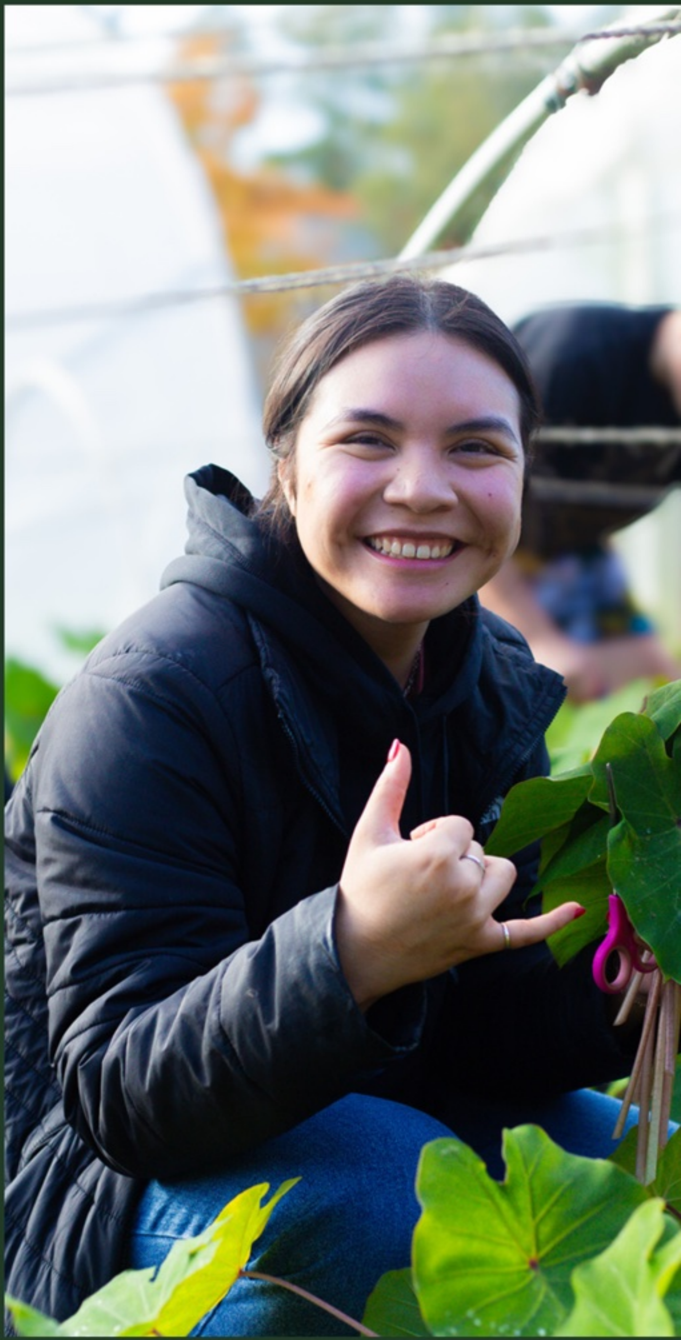

Results

Demographics

Twelve volunteers and five māla kalo leaders (17 individuals in total) completed the survey and interview. Of the 17 participants, three quarters were Female, nearly 40% were Native Hawaiian (41.2%) and over half had some college education. All participants reported having steady access to food, however, over half of the participants only ate traditional Native Hawaiian or Pacific Islander foods 1-2 times in the last month.

Feedback about the māla kalo

The majority of participants volunteered for one to two workdays, with almost 20% volunteering three to four days. Participants indicated they wanted to participate more frequently. All participants were satisfied with their experience at the māla kalo and all program leaders were extremely satisfied. Over half of the volunteers received a product grown in the māla kalo and shared the traditional foods prepared from the māla kalo with someone else.

Participants primary ethnicity

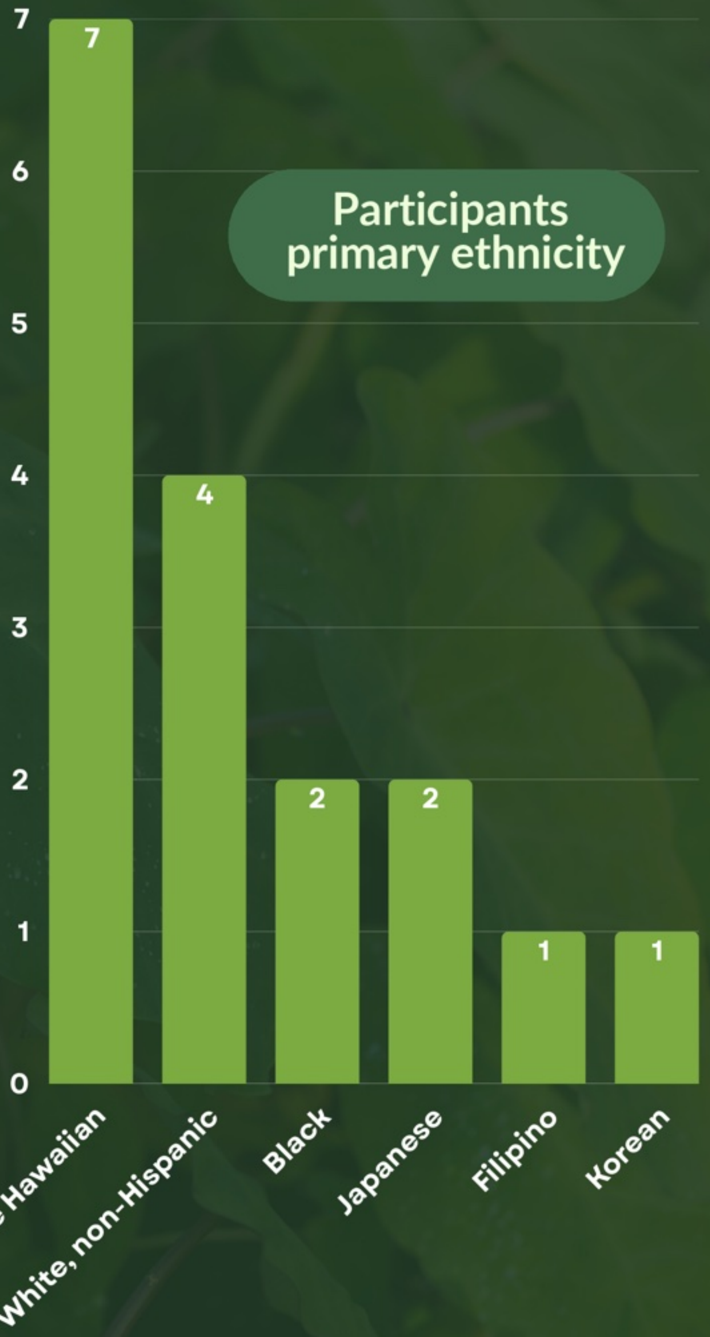

Education Level

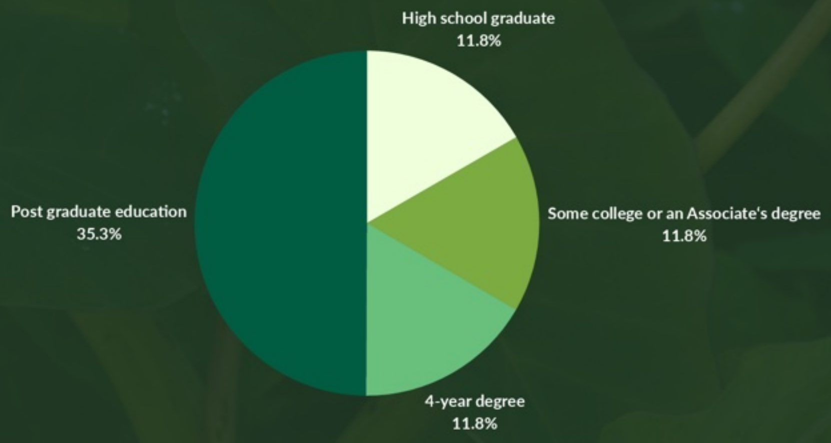

Gender

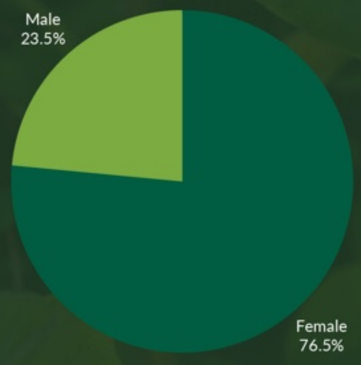

Benefits of the Māla Kalo

Participants shared that the most important benefit of a māla kalo is developing and building pili or relationships. Specifically, participants identified the following six benefits of the māla kalo which are exemplified by the following quotes.

Theme	*Volunteer or Program Leader Quotes*
Being together as a community in diaspora and connecting to land	It's the connection, the reconnection to land. I think, for Pacific Islander people we are innately connected to the land like there is this unspoken understanding of this connection we have to land without even being a farmer, like none of us have to be. Somehow, you know, 18 generations ago my first kupuna [elder] was connected to the land and of the land or whatever. And so, it's automatic. I think our community finds a sense of peace. Just being in a place and putting their hands in the soil, and a reminder that no matter where you live. We are still people of the land, so you don't have to be in Hawai'i to know the soil
Learning about and learning from kalo	What I've discovered in working with kalo the plant, I understand what kalo can teach us, because it is the most amazing plant. I don't wanna just call it a plant. It's the story of Hāloa, really, and learning the story of Hāloa really personifies kalo it. It gives it a different for me it looks different, and I feel different about it, because I know this the moʻolelo (story)...as you get older, like me, it takes on a whole different meaning. I don't wanna use the word resilience, I wanna use the word strength. Resilience has a sort of a 'woe is me' kind of connotation, in a way, but it's more strength. I'm gonna maybe more survive and have strength. And kalo the story of Hāloa and watching these plants grow here. You know, I made 45 laulau, I mean. you know that that'll take me almost a whole year to eat, but it's great! It provides. It's what the story says it provides for us, it provides for our meals, and it gives us incredible nutrition. You know how nutritious the belief is and working, it's also creative and flexible. So a laulau doesn't have to have a piece of pork in it. It can have anything and I've been thinking about different ways to make a better vegetarian laulau and I saw one that had broccoli and carrots in, and I went there's gotta be, there's gotta be a different way...
Having a safe space or puʻuhonua	I think the mala here just reminds me. of what I grew up with right in Hawaiʻi and so when you're in Hawaiʻi, [we] take everything for granted that is just surrounding you. And then, when you get to the continent, you realize it's not the same. And so you have to look for it. And having these spaces, intentional māla that are built for these purposes of community and connection. It reminds me for example, back to my own ʻāina where I grew up right when I see the taro patch. That's exactly like the view I had out my backyard as a young child. It brings me back to their brings good memories of my family, and and then feeling like my feet, are actually back in my native soil. Where it then brings out of me the desire to share much more about my culture and what I had learned as a child. And some of the things that were instilled in me from a child.

Benefits of the Māla Kalo

Participants shared that the most important benefit of a māla kalo is developing and building pili or relationships. Specifically, participants identified the following six benefits of the māla kalo which are exemplified by the following quotes.

Theme	***Volunteer or Program Leader Quotes***
Kaʻanalike ʻike (learning and sharing knowledge)	One thing that really came to me was how important it was for families to be there together. I think I saw a lot of different ages at the māla. I loved seeing like the elders and and the youth together and seeing that intergenerational knowledge exchange happen. Kind of reminds me of when my grandparents and my uncles were in the garden with me when I was younger. And I think it's a really powerful experience. Actually, because when you're a kid. You don't really think a lot about it. You're just like okay, yeah, yeah. I get it like snip here. Don't. Be careful here, whatever like. You know, just kind of not saying, all kids are like this, but for me at least. It was just like a Oh, yeah, this is neat but when you get a little bit older. You realize, just like how special that that experience was.
Perpetuating cultural practices and protocols	I want to instill these cultural protocols, values, experiences for my children growing up here on the continent so far away from our homeland...I need to teach them. And that's another important piece of coming to the māla or other cultural events like this is because is that resurgence that re-teaching of culture to the next generation that it's kind of been lost. I think about my dad, who has never, I don't think, worked in or been in a kalo field, and he grew up in Hawai'i. And when I talk to him about these experiences, when I show him pictures that I've taken, I can tell that sometimes tears fill, you know, fill his eyes, and he he's very emotional about how proud he is that this is this is happening, and especially how exciting that it's happening here on the continent in the Pacific Northwest.
Building a sense of identity and what it means to be Hawaiian on the continent	I was physically trying to act like I was back at 18, as everyone's working, working really hard. But I turned around and I saw 2 ladies sitting, and I didn't sit at all, and I saw them sitting, but I saw what they were doing with the students as they were sorting the rocks into different sizes, and they were sitting there with the stick pointing. You know the kid would come up and show the elder. The students had to say what the rock was before they could put it in the pile, and if they tried to put it in before they said it. I thought ahh, I should be taking my teaching skills and doing it that way because the the rest, the building on the wall was taken care of by the young dads and the young moms and the teens. When that happens, I need to extract myself and think of how I can participate as an elder, as a supporter, or bring water or pat people on the back. So I learned about that elder role... And I've been learning that, especially in the club, how it's important to be there, and elders to be there and to be seen, as we, you know, in Hawai'i we have.

Benefits of the Māla Kalo

Indigenous Connectedness Framework

These themes aligned with the Indigenous Connectedness Framework, centering relationships to ʻāina, moʻo (lineage), ʻohana (family), and kaiaulu (community), which are represented in the following conceptual model. Taken together, the māla kalo created more than a space to grow traditional foods for a community living in diaspora. The māla kalo brought the community together to build relationships, learn, and connect with the land away from "home."

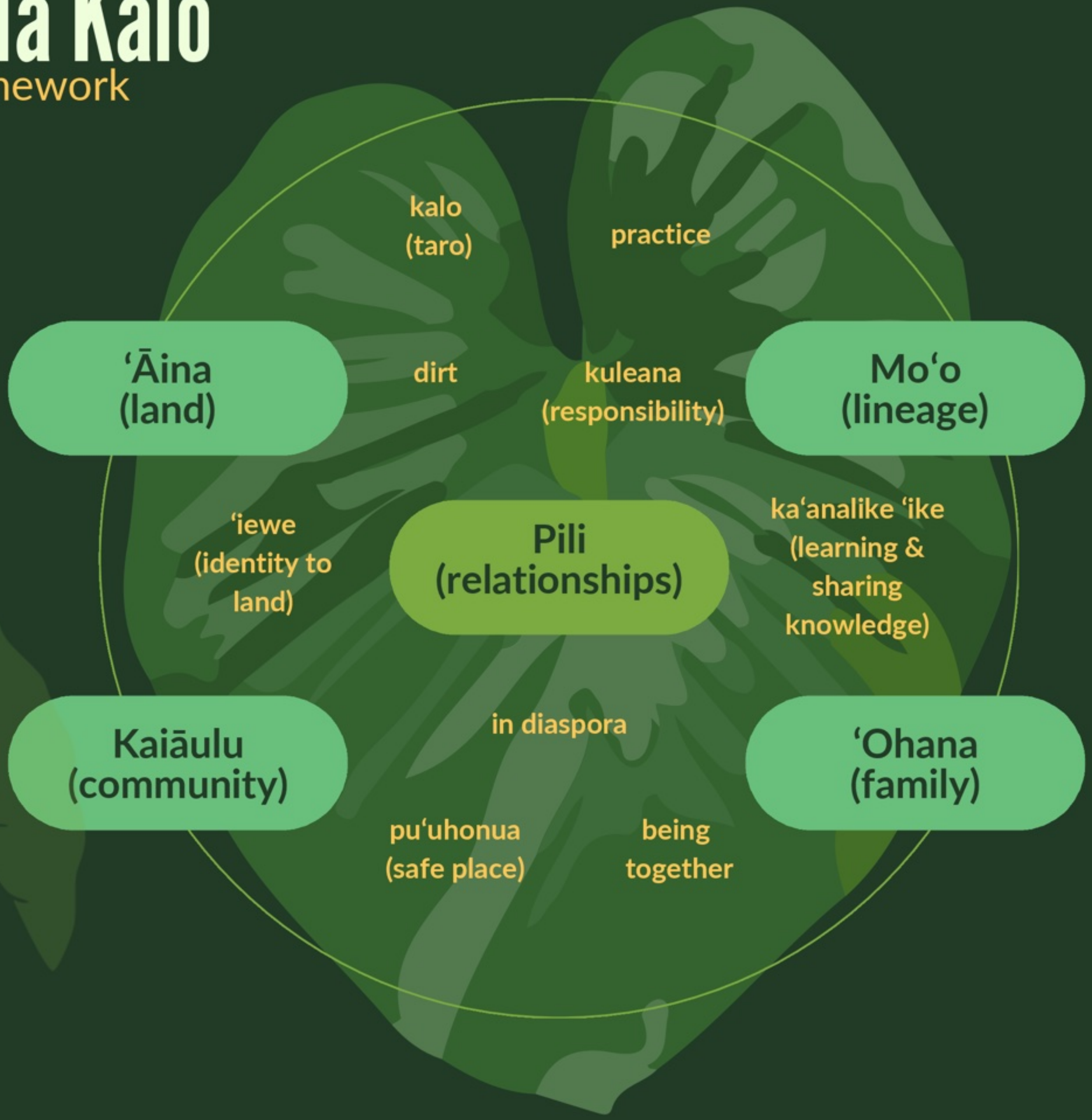

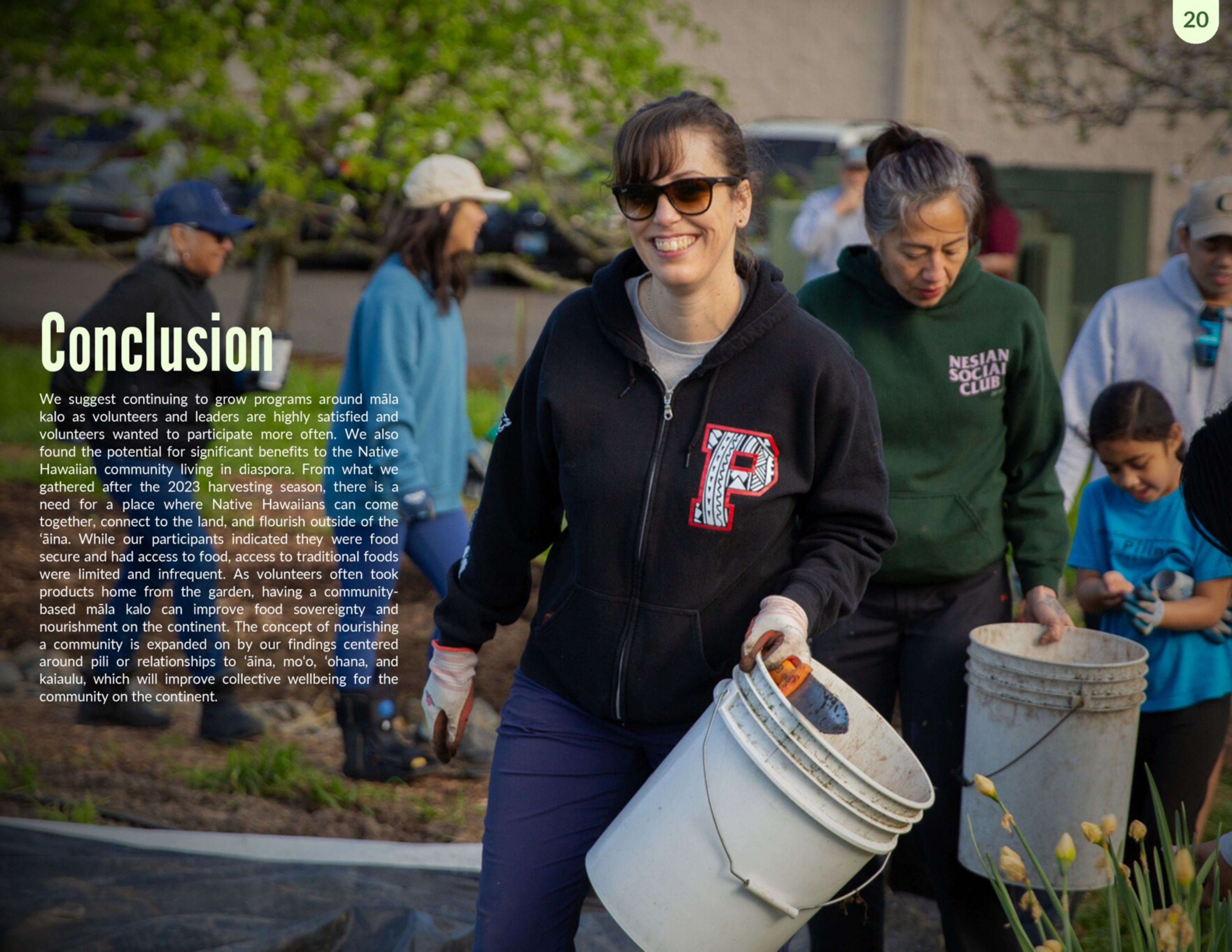

Conclusion

We suggest continuing to grow programs around māla kalo as volunteers and leaders are highly satisfied and volunteers wanted to participate more often. We also found the potential for significant benefits to the Native Hawaiian community living in diaspora. From what we gathered after the 2023 harvesting season, there is a need for a place where Native Hawaiians can come together, connect to the land, and flourish outside of the ʻāina. While our participants indicated they were food secure and had access to food, access to traditional foods were limited and infrequent. As volunteers often took products home from the garden, having a community-based māla kalo can improve food sovereignty and nourishment on the continent. The concept of nourishing a community is expanded on by our findings centered around pili or relationships to ʻāina, moʻo, ʻohana, and kaiaulu, which will improve collective wellbeing for the community on the continent.

Moving Forward

Since starting the māla kalo, communities and individuals around us have reached out to KALO HCC to learn how to start their own māla kalo. As such, we will continue to build resources for other community organizations to start similar programs.

We will continue to cultivate the Piko to Piko program, including:

- Identifying carbon-neutral greenhouses to grow kalo throughout the year
- Expand partnerships to raise awareness of growing kalo on the continent and continue to reach and engage the Native Hawaiian community
- Refine the resources to support the māla kalo including a growing guide, ʻohana program, and assistance for other organizations on the continent to develop māla kalo
- Apply for additional funding to support the māla kalo, hui, and future programming

He aliʻi ka ʻāina; He kauā ke kanaka.
The land is chief; People are its servant.

ʻŌlelo Noʻeau #531
